# Supplementary material for: Biophysics-based protein language models for protein engineering
Source: bioRxiv. 2025 Jan 14:2024.03.15.585128. Originally published 2024 Mar 17. Preprint. [Version 2] doi: 10.1101/2024.03.15.585128 (PMC10980077; doi:10.1101/2024.03.15.585128)
Supplement: Supplementary file 1 [file NIHPP2024.03.15.585128v2-supplement-1.pdf]

bioRxiv preprint doi: https://doi.org/10.1101/2024.03.15.585128; this version posted January 14, 2025. The copyright holder for this preprint (which was not certified by peer review) is the author/funder, who has granted bioRxiv a license to display the preprint in perpetuity. It is made available under aCC-BY 4.0 International license.

Supplementary Information: Biophysics-based protein language models for protein engineering

Sam Gelman, Bryce Johnson, Chase Freschlin, Arnav Sharma, Sameer D'Costa, John Peters, Anthony Gitter, Philip A. Romero

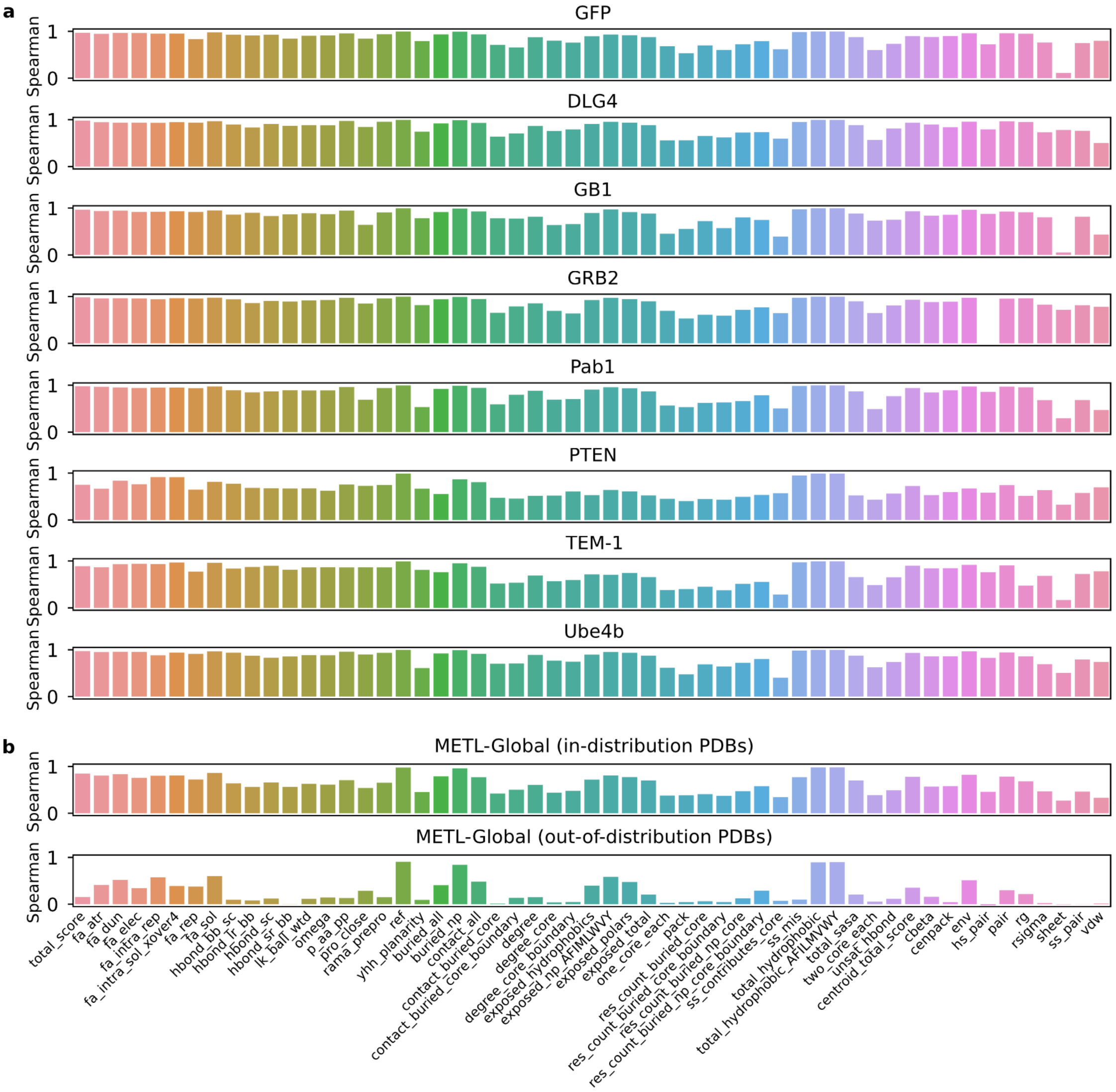

Figure S1. **Performance of pretrained METL source models in predicting Rosetta scores.** This figure shows Spearman correlations between true and predicted Rosetta scores for each of the 55 Rosetta score terms. (a) Correlation of METL-Local models predicting Rosetta biophysical attributes for the protein-specific pretraining data test sets. (b) Correlation of METL-Global in predicting Rosetta scores for protein variants originating from in-distribution base PDBs (those included in METL-Global pretraining) and out-of-distribution base PDBs (those not included). We show the mean Spearman correlation across base PDBs. To evaluate in-distribution PDBs, we used variants in the pretraining data test set. To evaluate out-of-distribution PDBs, we used variants from the deep mutational scanning datasets included in this study. METL-Global performs substantially better for in-distribution PDBs, suggesting there is overfitting to the PDBs present in the training data.

bioRxiv preprint doi: https://doi.org/10.1101/2024.03.15.585128; this version posted January 14, 2025. The copyright holder for this preprint (which was not certified by peer review) is the author/funder, who has granted bioRxiv a license to display the preprint in perpetuity. It is made available under aCC-BY 4.0 International license.

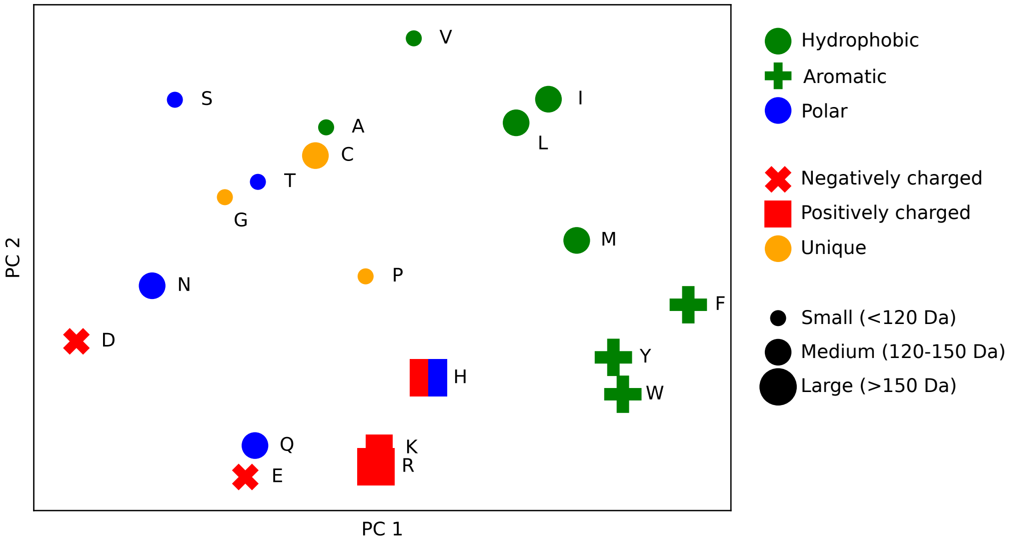

Figure S2. METL-Global amino acid embeddings We applied principal component analysis (PCA) to reduce the METL-Global length 512 amino acid embeddings down to 2 dimensions, capturing 33% of the variance in data. This scatter plot of the 2-dimensional amino acid embeddings is annotated with amino acid properties. METL-Global groups amino acids with similar biochemical properties in the embedding space, like protein language models (PLMs) trained on millions of natural protein sequences [1].

bioRxiv preprint doi: https://doi.org/10.1101/2024.03.15.585128; this version posted January 14, 2025. The copyright holder for this preprint (which was not certified by peer review) is the author/funder, who has granted bioRxiv a license to display the preprint in perpetuity. It is made available under aCC-BY 4.0 International license.

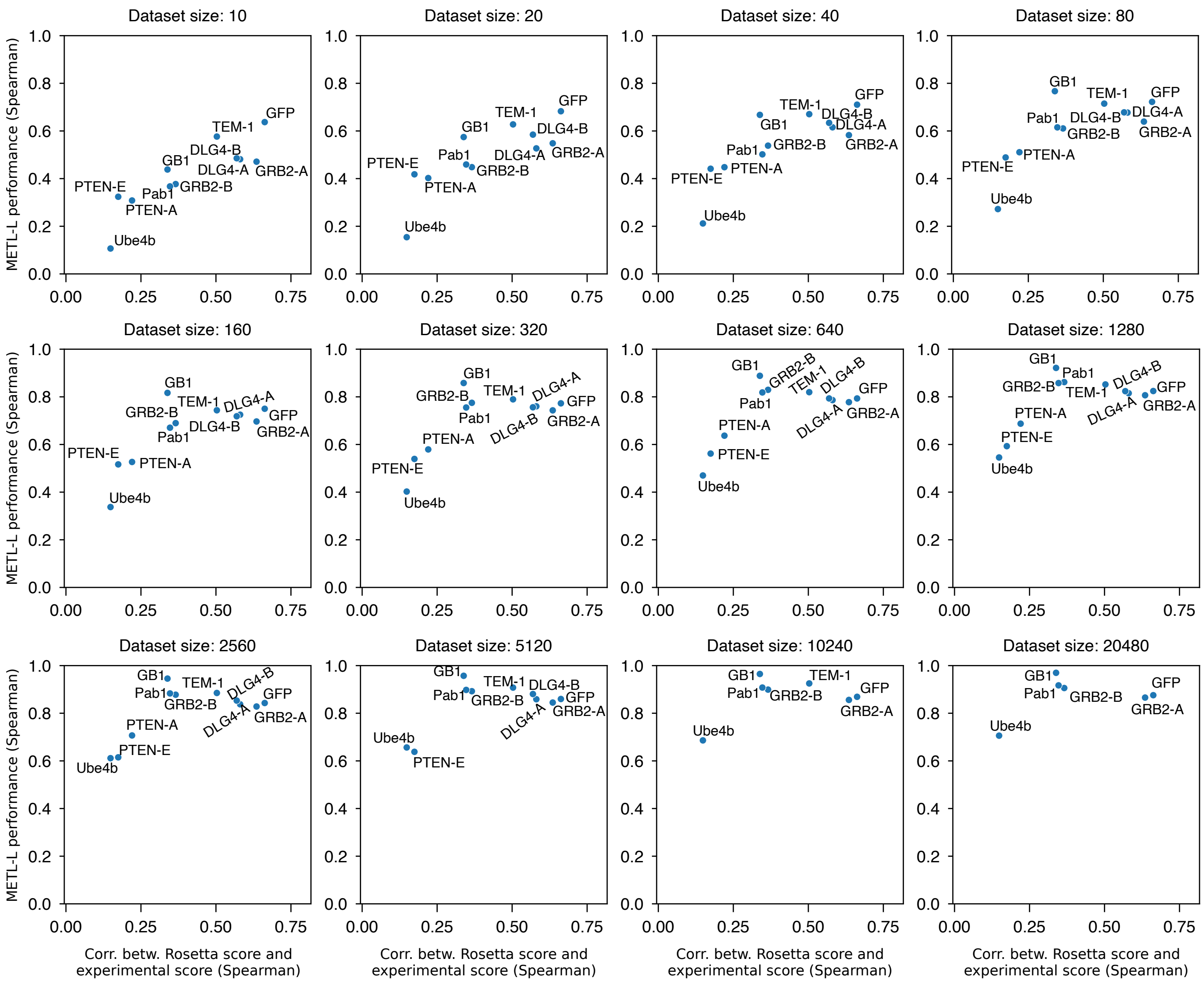

Figure S3. **Relationship between METL-Local performance and the relatedness of Rosetta and experimental scores.** The figure displays a series of scatterplots showing the relationship between METL-Local performance and the relatedness of Rosetta and experimental scores, across multiple experimental datasets and training set sizes. The x-axis shows the Spearman correlation between Rosetta total score and the experimental functional score for the entire dataset, representing the similarity between the Rosetta total score and the experimental functional score. The y-axis shows the METL-Local performance for the respective training set size, as determined by the Spearman correlation on the test set. As the similarity between Rosetta total score and the experimental functional score increases, so does the METL-Local performance, at least for small training set sizes. However, with increasing experimental training set sizes, the similarity between Rosetta total score and experimental functional score becomes less important to the METL-Local performance, suggesting a shift in METL-Local away from the Rosetta pretraining data and more toward the experimental finetuning data.

bioRxiv preprint doi: https://doi.org/10.1101/2024.03.15.585128; this version posted January 14, 2025. The copyright holder for this preprint (which was not certified by peer review) is the author/funder, who has granted bioRxiv a license to display the preprint in perpetuity. It is made available under aCC-BY 4.0 International license.

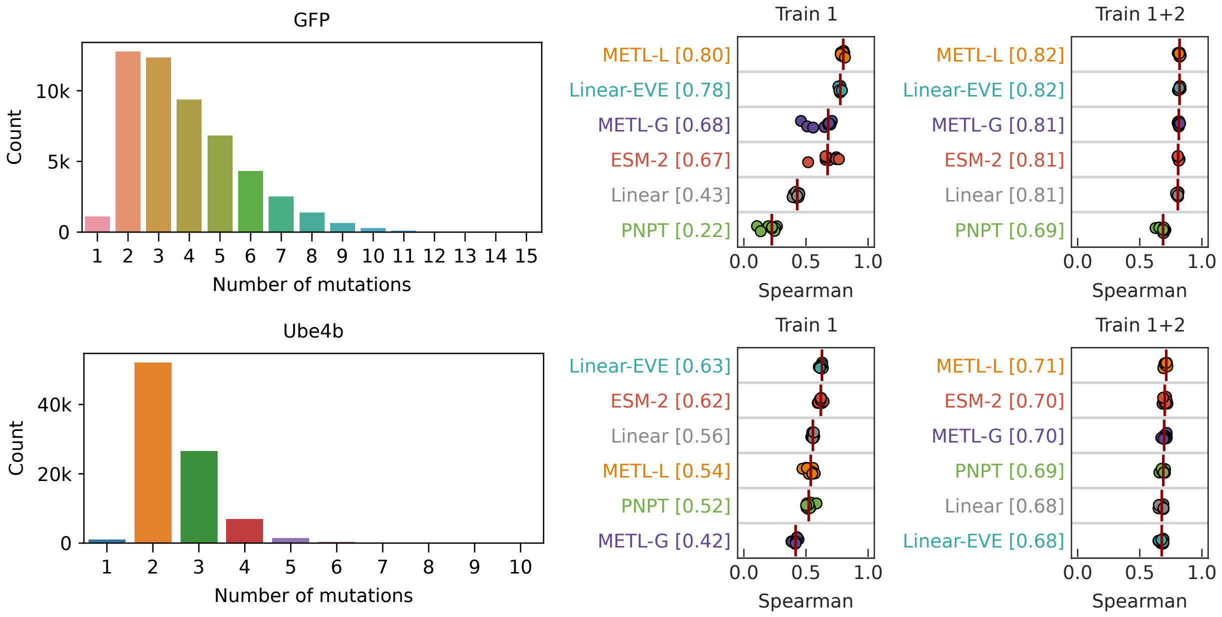

Figure S4. Regime extrapolation for GFP and Ube4b datasets. The GFP and Ube4b datasets contain variants with higher order mutations, enabling us to test two types of regime extrapolation: Train 1 and Train 1+2. The bar plots (left) show the counts of variants with the specified number of mutations for each dataset. The strip plots (right) show the performance of regime extrapolation for Train 1, where we train on single substitution variants and evaluate on variants with 2+ substitutions, and Train 1+2, where we train on variants with single or double substitutions, and evaluate on variants with 3+ substitutions. The strip plots show the performance of 9 test set replicates, and the red vertical line denotes the median.

bioRxiv preprint doi: https://doi.org/10.1101/2024.03.15.585128; this version posted January 14, 2025. The copyright holder for this preprint (which was not certified by peer review) is the author/funder, who has granted bioRxiv a license to display the preprint in perpetuity. It is made available under aCC-BY 4.0 International license.

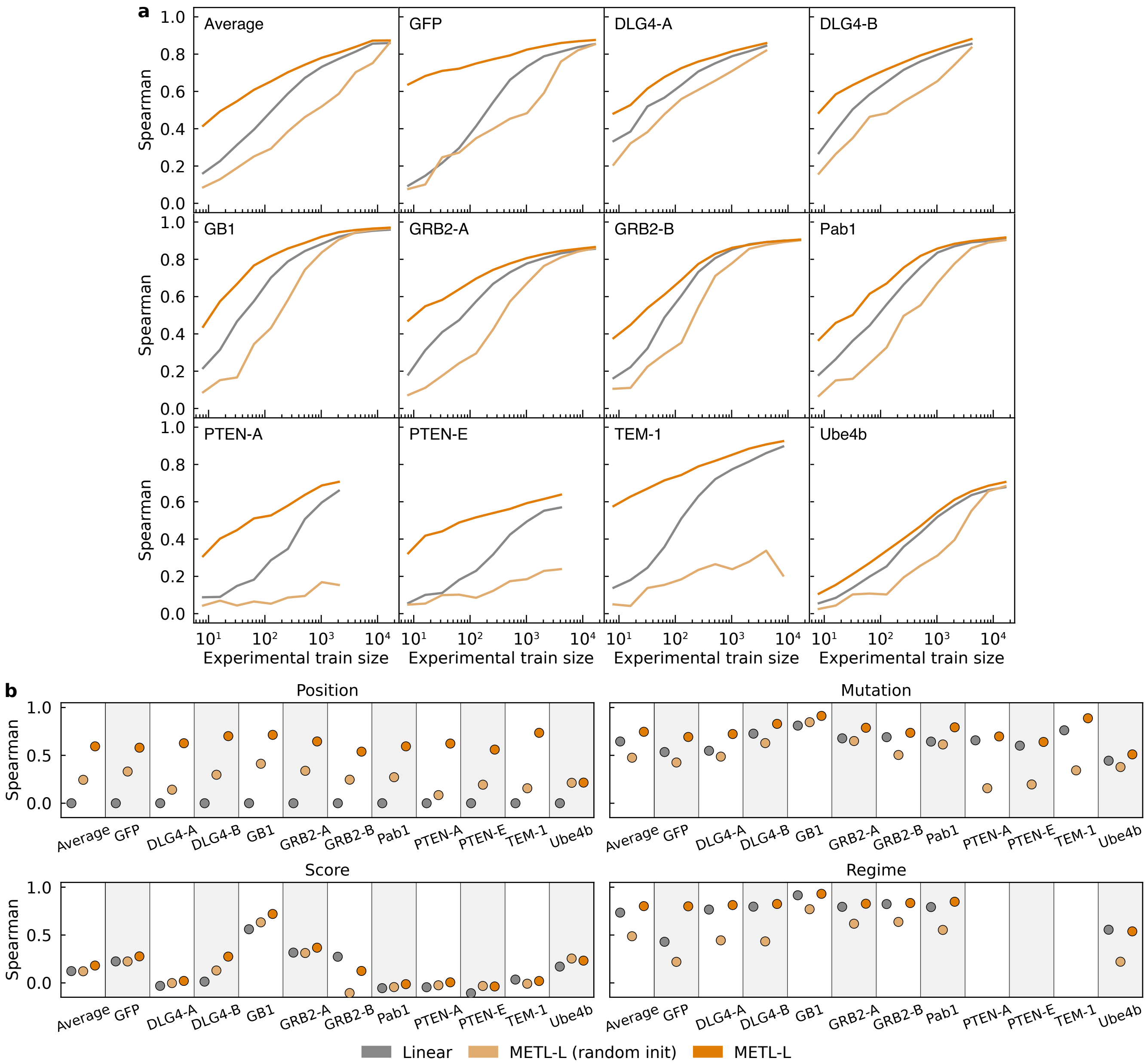

Figure S5. Performance of METL-Local with and without pretraining. These plots show the correlation performance of Linear, METL-Local (random init), and METL-Local. METL-Local (random init) is a model with the same architecture as METL-Local but without pretraining on Rosetta scores. (a) The learning curves show that METL-Local (random init) substantially underperforms both Linear and pretrained METL-Local, emphasizing the impact pretraining on Rosetta scores has on this transformer-based architecture. Given enough experimental training data, METL-Local (random init) converges to the performance of the other models for most datasets. (b) METL-Local (random init) outperforms Linear for position extrapolation due to the fact that Linear is not able to perform position extrapolation but is substantially worse than METL-Local. For the other types of extrapolation, METL-Local (random init) performs about the same or worse than Linear.

bioRxiv preprint doi: https://doi.org/10.1101/2024.03.15.585128; this version posted January 14, 2025. The copyright holder for this preprint (which was not certified by peer review) is the author/funder, who has granted bioRxiv a license to display the preprint in perpetuity. It is made available under aCC-BY 4.0 International license.

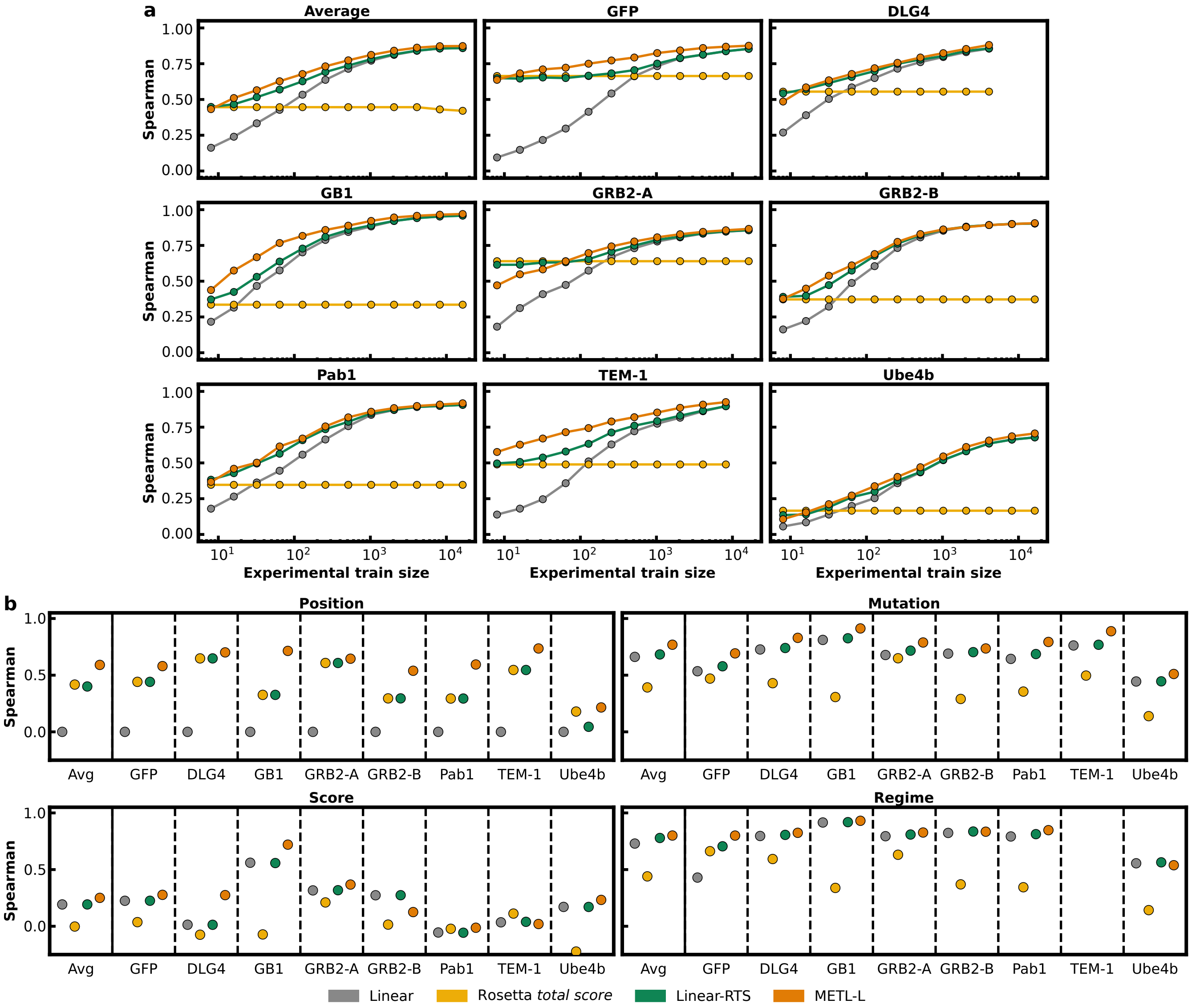

Figure S6. Performance of baseline models directly using Rosetta total score. Rosetta *total score* is the score term from Rosetta with no supervised training on experimental data. Linear-RTS is a linear ridge regression model trained on experimental data with one hot encoding features augmented with the Rosetta *total score* as an additional input feature. Both of these models require running Rosetta to compute the *total score* for every variant, even during inference. For comparison, this figure also shows the performance of Linear and METL-Local. (a) For small training set sizes, incorporating Rosetta *total score* as an additional input feature for ridge regression greatly improved performance over solely using one hot encoding features, as demonstrated by the difference in performance between Linear and Linear-RTS. While Linear-RTS sometimes matched METL-Local's performance and even exceeded it on the GRB2-A dataset, METL-Local still outperformed Linear-RTS on average by a small amount and is much faster. (b) METL-Local outperformed Linear-RTS across most datasets and extrapolation tasks. The performance differences were sometimes substantial, such as for position extrapolation with GB1. In other cases, the performance differences were much smaller, such as for regime extrapolation. This analysis does not use all of the deep mutational scanning datasets.

bioRxiv preprint doi: https://doi.org/10.1101/2024.03.15.585128; this version posted January 14, 2025. The copyright holder for this preprint (which was not certified by peer review) is the author/funder, who has granted bioRxiv a license to display the preprint in perpetuity. It is made available under aCC-BY 4.0 International license.

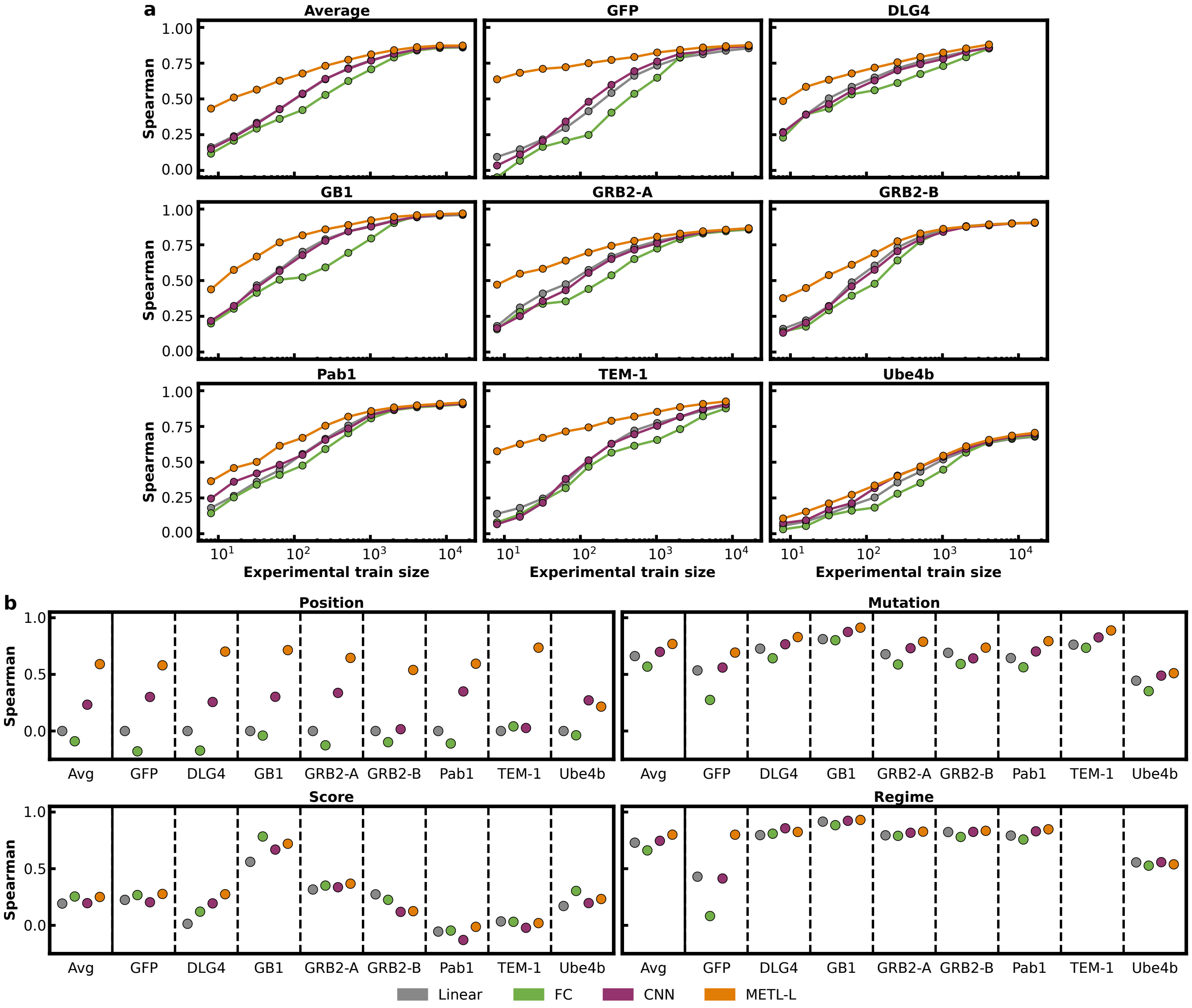

Figure S7. **Performance of additional baseline models.** Correlation performance of Linear, fully-connected networks (FC), sequence convolutional networks (CNN), and METL-Local. (a) METL-Local has strong advantages over the fully-connected network and CNN on nearly every dataset. The CNN performed about the same as Linear across different sized training sets. The fully-connected network typically performed about the same or worse than Linear, especially for mid-size training sets. (b) METL-Local exhibits much better position extrapolation capabilities than all three baseline models as well as substantially better GFP regime extrapolation. This analysis does not use all of the deep mutational scanning datasets.

bioRxiv preprint doi: https://doi.org/10.1101/2024.03.15.585128; this version posted January 14, 2025. The copyright holder for this preprint (which was not certified by peer review) is the author/funder, who has granted bioRxiv a license to display the preprint in perpetuity. It is made available under aCC-BY 4.0 International license.

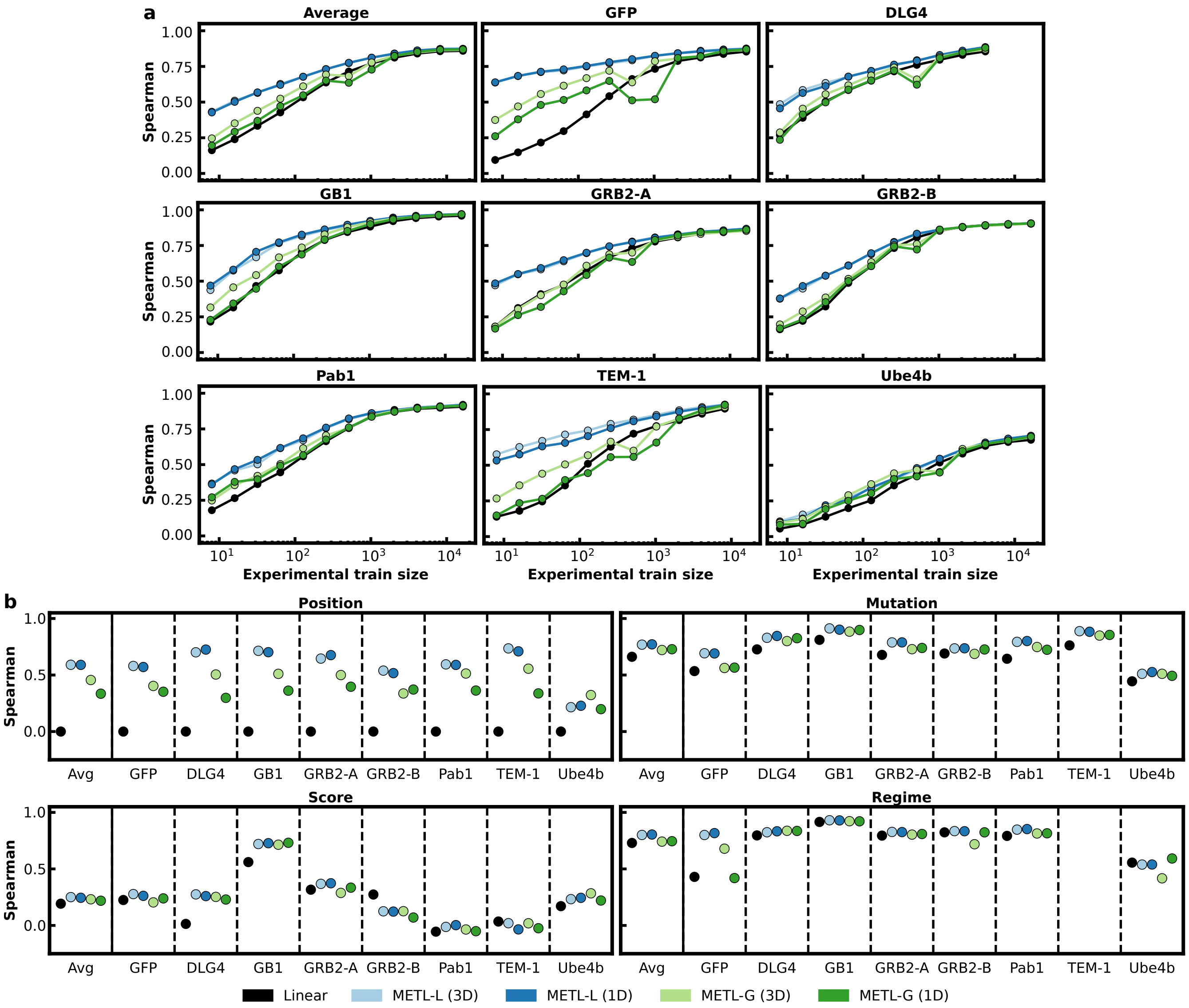

Figure S8. Performance of one-dimensional and three-dimensional relative position embeddings. This figure shows the performance of METL-Local and METL-Global with one-dimensional (1D) sequence-based and three-dimensional (3D) structure-based relative position embeddings. (a) Learning curves showing Spearman correlation between true and predicted scores across a range of training set sizes. (b) Spearman correlation between true and predicted scores for position, mutation, score, and regime extrapolation. Overall, METL-Local does not benefit much from three-dimensional embeddings over one-dimensional, while METL-Global shows consistent improvement with the three-dimensional embeddings. This analysis does not use all of the deep mutational scanning datasets.

bioRxiv preprint doi: https://doi.org/10.1101/2024.03.15.585128; this version posted January 14, 2025. The copyright holder for this preprint (which was not certified by peer review) is the author/funder, who has granted bioRxiv a license to display the preprint in perpetuity. It is made available under aCC-BY 4.0 International license.

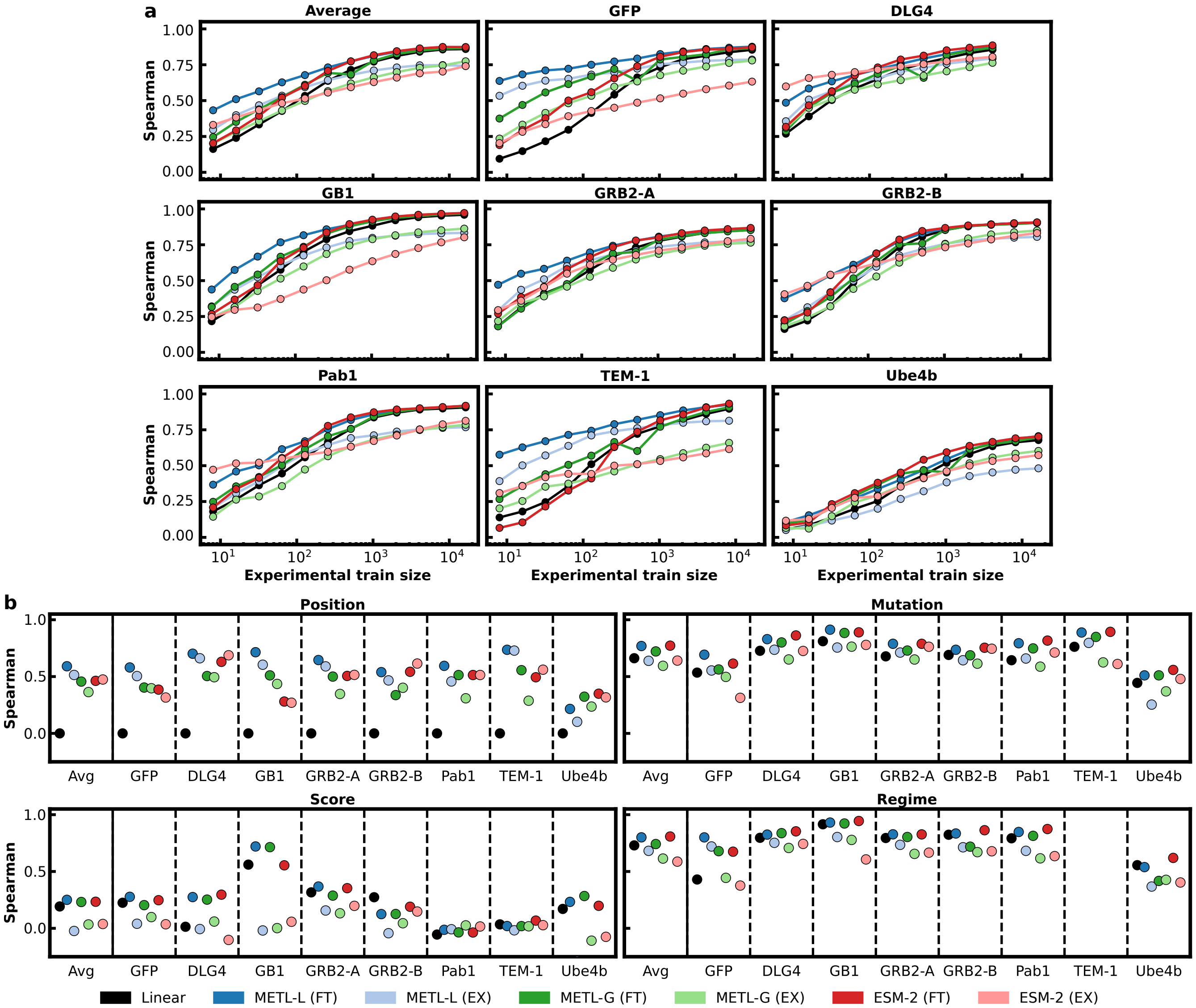

Figure S9. Performance of PLM finetuning and feature extraction. This figure shows the performance of METL-Local, METL-Global, and ESM-2 with both finetuning (FT) and feature extraction (EX). To perform feature extraction, we saved outputs from the appropriate internal layer of each model and then used those features as inputs to train linear ridge regression. Finetuning consistently outperformed feature extraction for METL-Local and METL-Global across (a) different training set sizes and (b) extrapolation tasks. For ESM-2, there were several instances where feature extraction substantially outperformed finetuning when applied to (a) small training set sizes, namely for the DLG4, GRB2-B, Pab1, and TEM-1 datasets. Notably, the performance of ESM-2 feature extraction exceeded the performance METL-Local finetuning for DLG4 and Pab1 with the smallest training set sizes. For (b) extrapolation tasks, ESM-2 finetuning generally performed better than feature extraction. This analysis does not use all of the deep mutational scanning datasets.

bioRxiv preprint doi: https://doi.org/10.1101/2024.03.15.585128; this version posted January 14, 2025. The copyright holder for this preprint (which was not certified by peer review) is the author/funder, who has granted bioRxiv a license to display the preprint in perpetuity. It is made available under aCC-BY 4.0 International license.

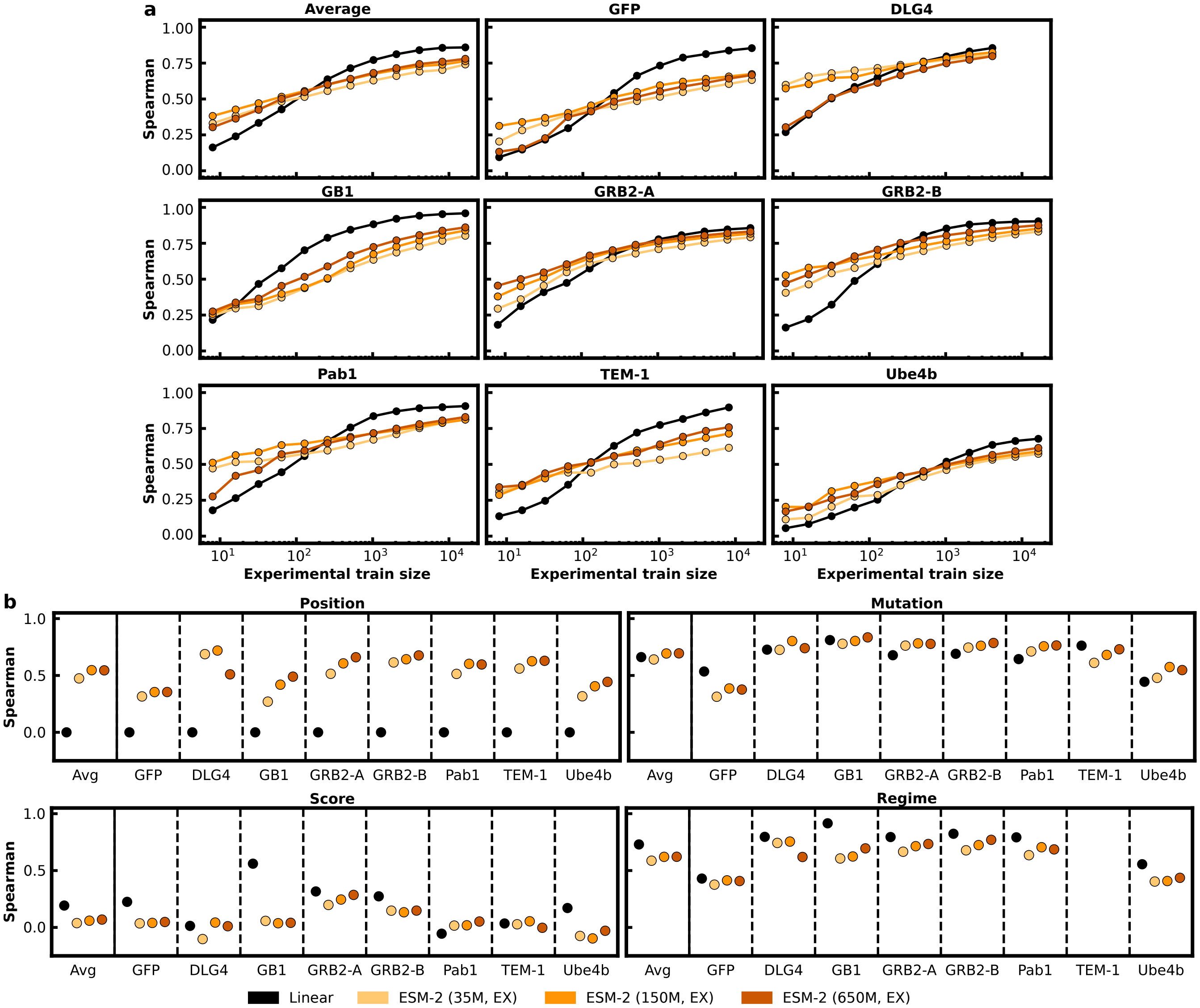

Figure S10. Feature extraction performance of ESM-2 models with 35M, 150M, and 650M parameters. (a) Across the range of training set sizes, the 150M parameter model consistently outperformed the 35M parameter model, with the exception of the DLG4 dataset, where the 35M parameter model performed better. Surprisingly, for small training set sizes, the 650M parameter model performed worse than both the 35M and 150M parameter models with the GFP, DLG4, and Pab1 datasets. For larger training set sizes, the 650M parameter model offered some improvement over the 35M and 150M parameter models with the GB1, GRB2-A, and GRB2-B datasets. However, in all cases Linear was the best model with larger datasets. (b) Across extrapolation tasks, the 35M parameter model tended to perform worse than the 150M and 650M parameter models. The 650M parameter model often performed the best, but not in all instances, and the differences between the models were minor in some cases. The Linear baseline was better than the feature extraction ESM-2 models on average for score and regime extrapolation. This analysis does not use all of the deep mutational scanning datasets.

bioRxiv preprint doi: https://doi.org/10.1101/2024.03.15.585128; this version posted January 14, 2025. The copyright holder for this preprint (which was not certified by peer review) is the author/funder, who has granted bioRxiv a license to display the preprint in perpetuity. It is made available under aCC-BY 4.0 International license.

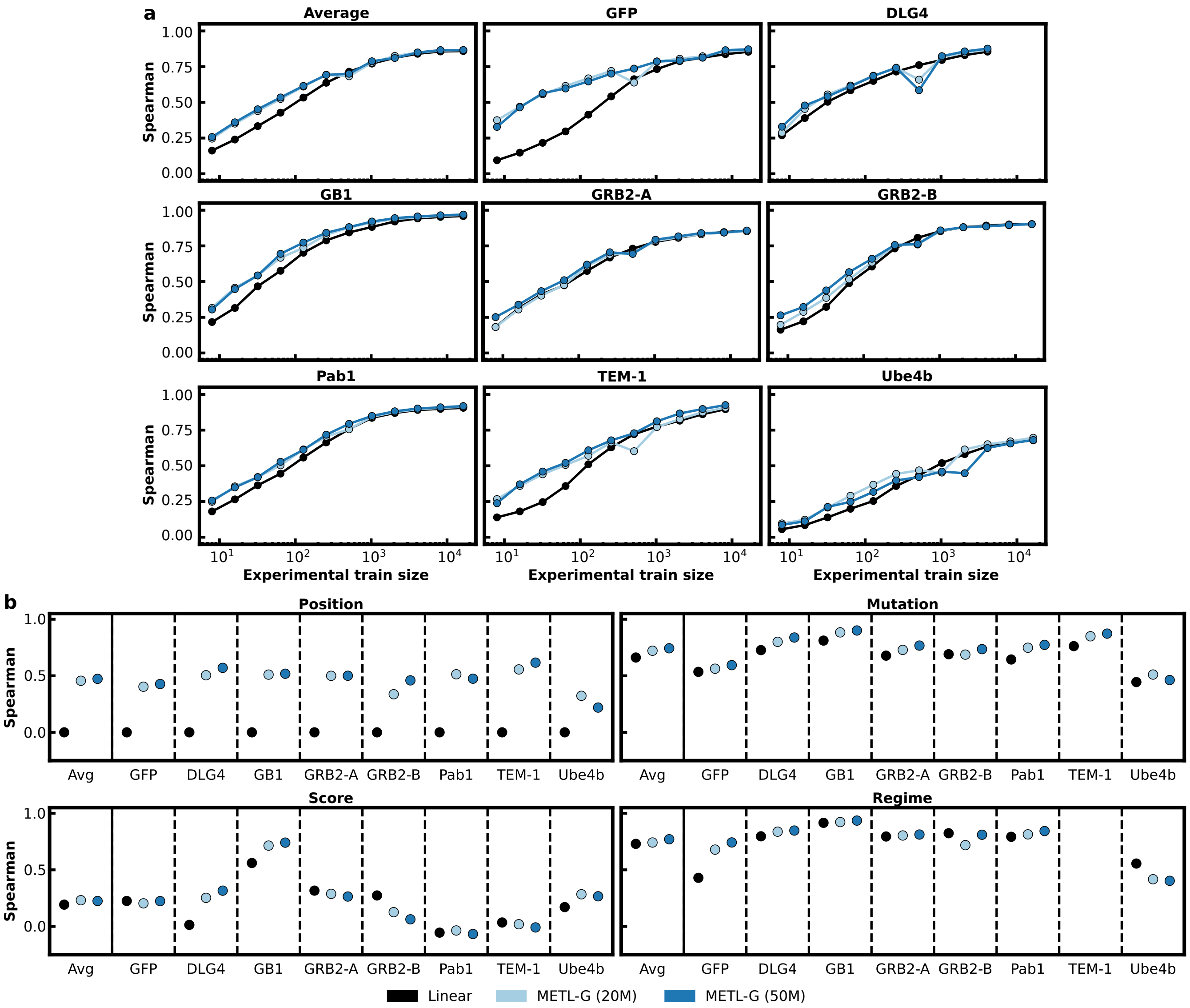

Figure S11. Performance of METL-Global with 20M and 50M parameters (a) Across different training set sizes, the 50M parameter model performed similarly to the 20M parameter model on average. (b) For position, mutation, and regime extrapolation, the 50M parameter model performed slightly better on average than the 20M parameter model. For score extrapolation, the two models performed similarly on average. This analysis does not use all of the deep mutational scanning datasets.

bioRxiv preprint doi: https://doi.org/10.1101/2024.03.15.585128; this version posted January 14, 2025. The copyright holder for this preprint (which was not certified by peer review) is the author/funder, who has granted bioRxiv a license to display the preprint in perpetuity. It is made available under aCC-BY 4.0 International license.

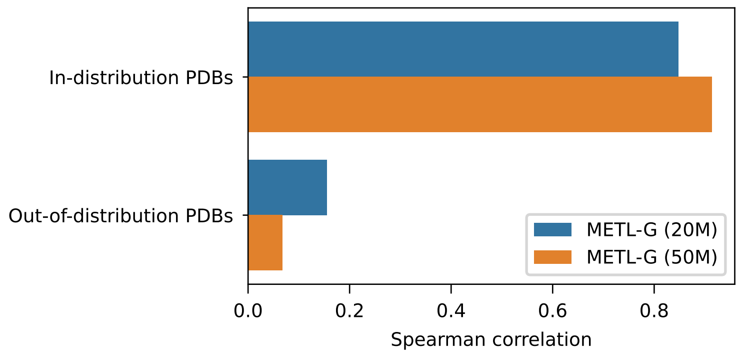

Figure S12. Performance of METL-Global source models predicting Rosetta *total score*. This figure shows the performance of 20M and 50M parameter METL-Global source models on predicting Rosetta *total score* for both in-distribution and out-of-distribution PDBs. In-distribution PDBs are the 148 PDBs that were used as part of the METL-Global pretraining data, while out-of-distribution PDBs consist of the experimental dataset PDBs, which were not used for METL-Global pretraining. The 50M parameter METL-Global model overfits more than the 20M parameter model when predicting Rosetta *total score* on in-distribution PDBs, and it generalizes worse to out-of-distribution PDBs.

bioRxiv preprint doi: https://doi.org/10.1101/2024.03.15.585128; this version posted January 14, 2025. The copyright holder for this preprint (which was not certified by peer review) is the author/funder, who has granted bioRxiv a license to display the preprint in perpetuity. It is made available under aCC-BY 4.0 International license.

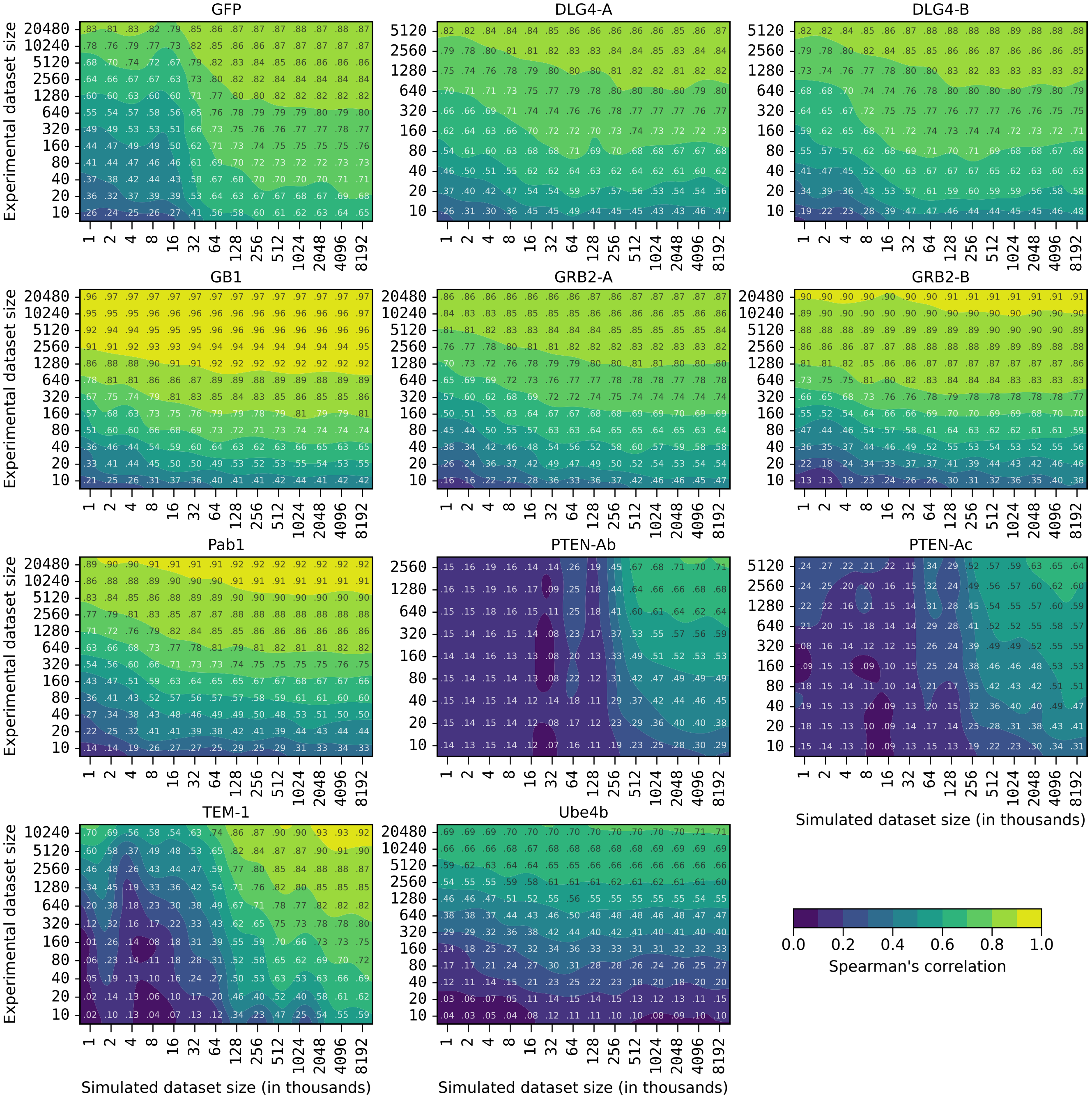

Figure S13. Relationships between experimental and simulated data quantities. These contour plots illustrate the test set Spearman's correlation resulting from training METL-Local with varying amounts of simulated (pretraining) and experimental (finetuning) data. The plots display a grid of Spearman's correlation values corresponding to discrete combinations of experimental and simulated dataset sizes.

bioRxiv preprint doi: https://doi.org/10.1101/2024.03.15.585128; this version posted January 14, 2025. The copyright holder for this preprint (which was not certified by peer review) is the author/funder, who has granted bioRxiv a license to display the preprint in perpetuity. It is made available under aCC-BY 4.0 International license.

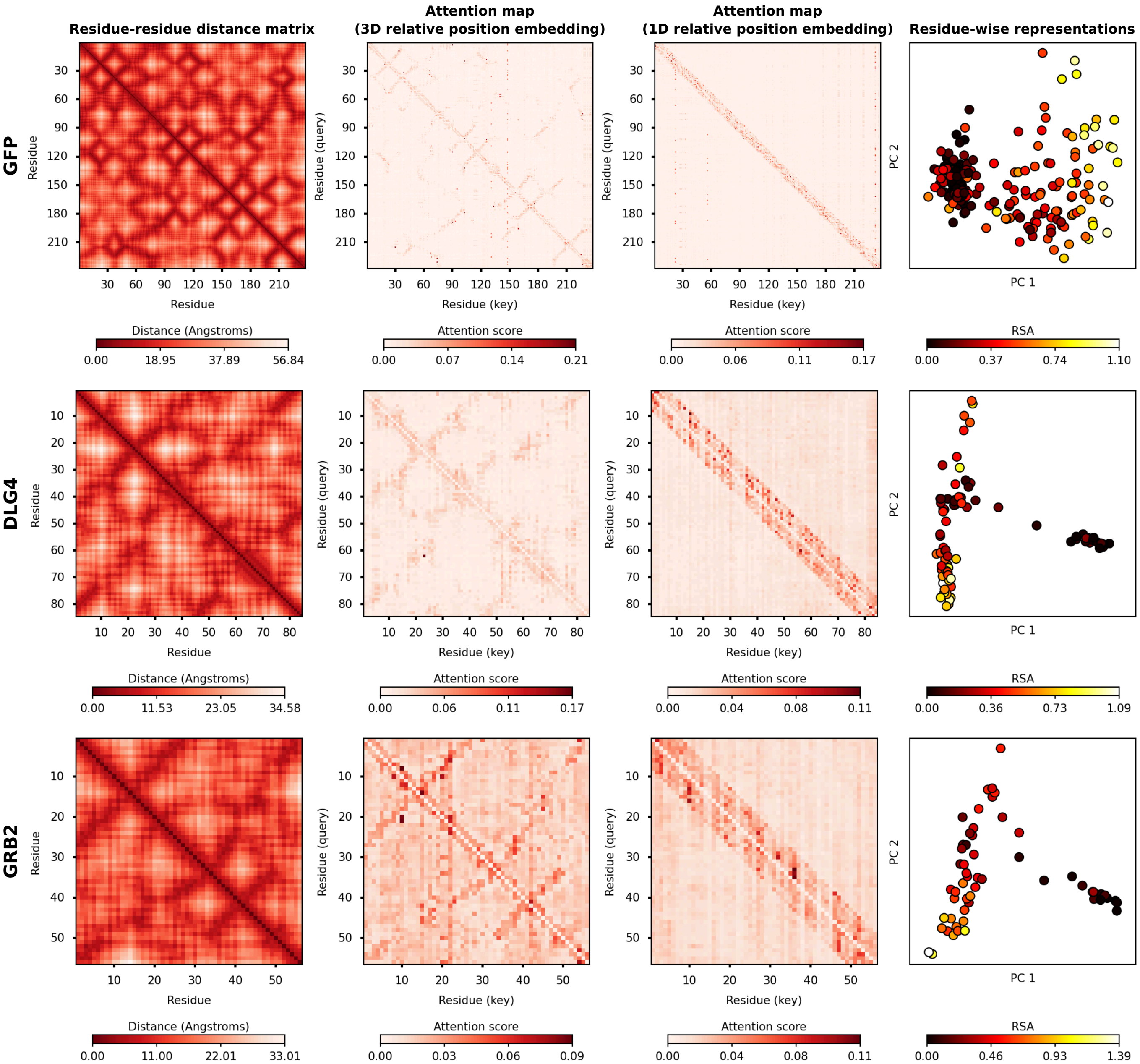

Figure S14. METL attention maps and residue representations for GFP, DLG4, and GRB2. The residue distance matrix shows Cβ distances between residues for the wild-type structure. The attention maps show the mean attention across layers and attention heads for the wild-type sequence when it is fed as input to the pretrained METL-Local model. The residue-wise representations show the principal component analysis (PCA) of the residue representations output by the pretrained METL-Local model, averaged across the 20 possible amino acids at each sequence position. Points are colored according to relative solvent accessibility (RSA) computed from the wild-type structure.

bioRxiv preprint doi: https://doi.org/10.1101/2024.03.15.585128; this version posted January 14, 2025. The copyright holder for this preprint (which was not certified by peer review) is the author/funder, who has granted bioRxiv a license to display the preprint in perpetuity. It is made available under aCC-BY 4.0 International license.

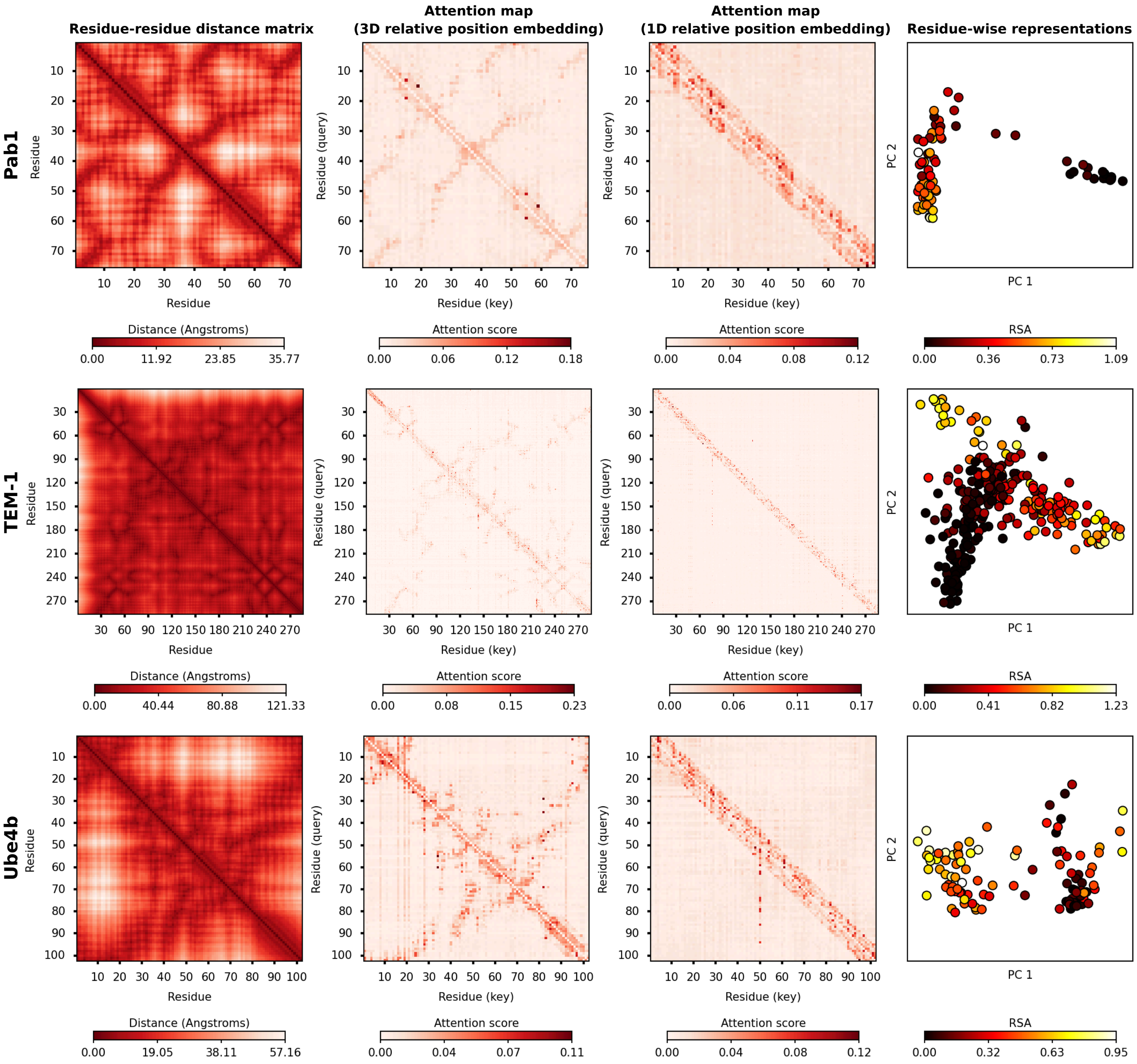

Figure S15. METL attention maps and residue representations for Pab1, TEM-1, and Ube4b. The residue distance matrix shows Cβ distances between residues for the wild-type structure. The attention maps show the mean attention across layers and attention heads for the wild-type sequence when it is fed as input to the pretrained METL-Local model. The residue-wise representations show the principal component analysis (PCA) of the residue representations output by the pretrained METL-Local model, averaged across the 20 possible amino acids at each sequence position. Points are colored according to relative solvent accessibility (RSA) computed from the wild-type structure.

bioRxiv preprint doi: https://doi.org/10.1101/2024.03.15.585128; this version posted January 14, 2025. The copyright holder for this preprint (which was not certified by peer review) is the author/funder, who has granted bioRxiv a license to display the preprint in perpetuity. It is made available under aCC-BY 4.0 International license.

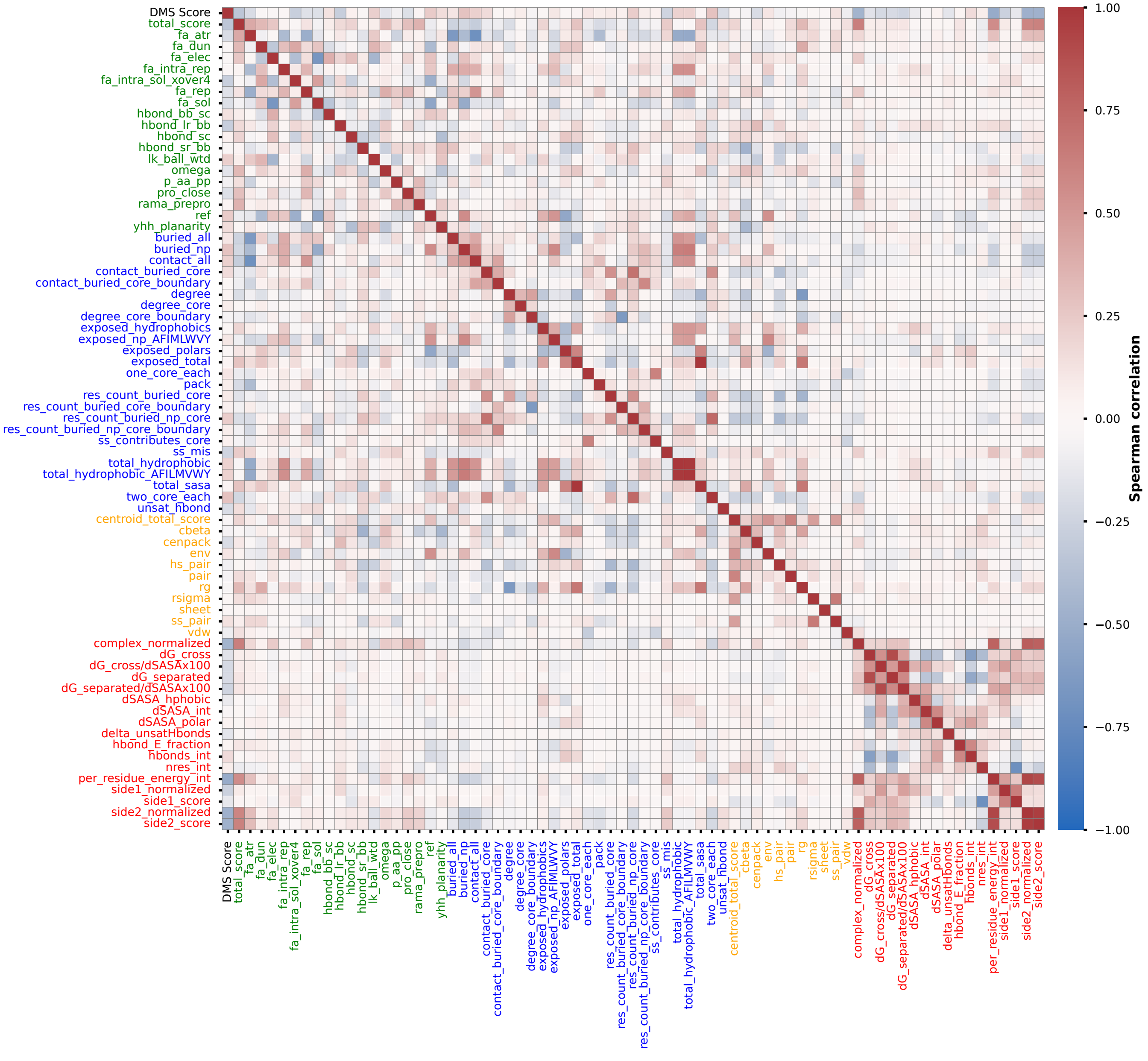

Figure S16. Pairwise correlations between GB1 DMS score and Rosetta scores. Heatmap showing pairwise Spearman correlations between the GB1 experimental functional score (DMS Score) and Rosetta score terms. Rosetta scores are color coded, with green representing all-atom REF15 scores, blue representing filter scores, orange representing centroid score3 scores, and red representing InterfaceAnalyzer binding scores. Correlations were computed using the GB1 DMS variants.

bioRxiv preprint doi: https://doi.org/10.1101/2024.03.15.585128; this version posted January 14, 2025. The copyright holder for this preprint (which was not certified by peer review) is the author/funder, who has granted bioRxiv a license to display the preprint in perpetuity. It is made available under aCC-BY 4.0 International license.

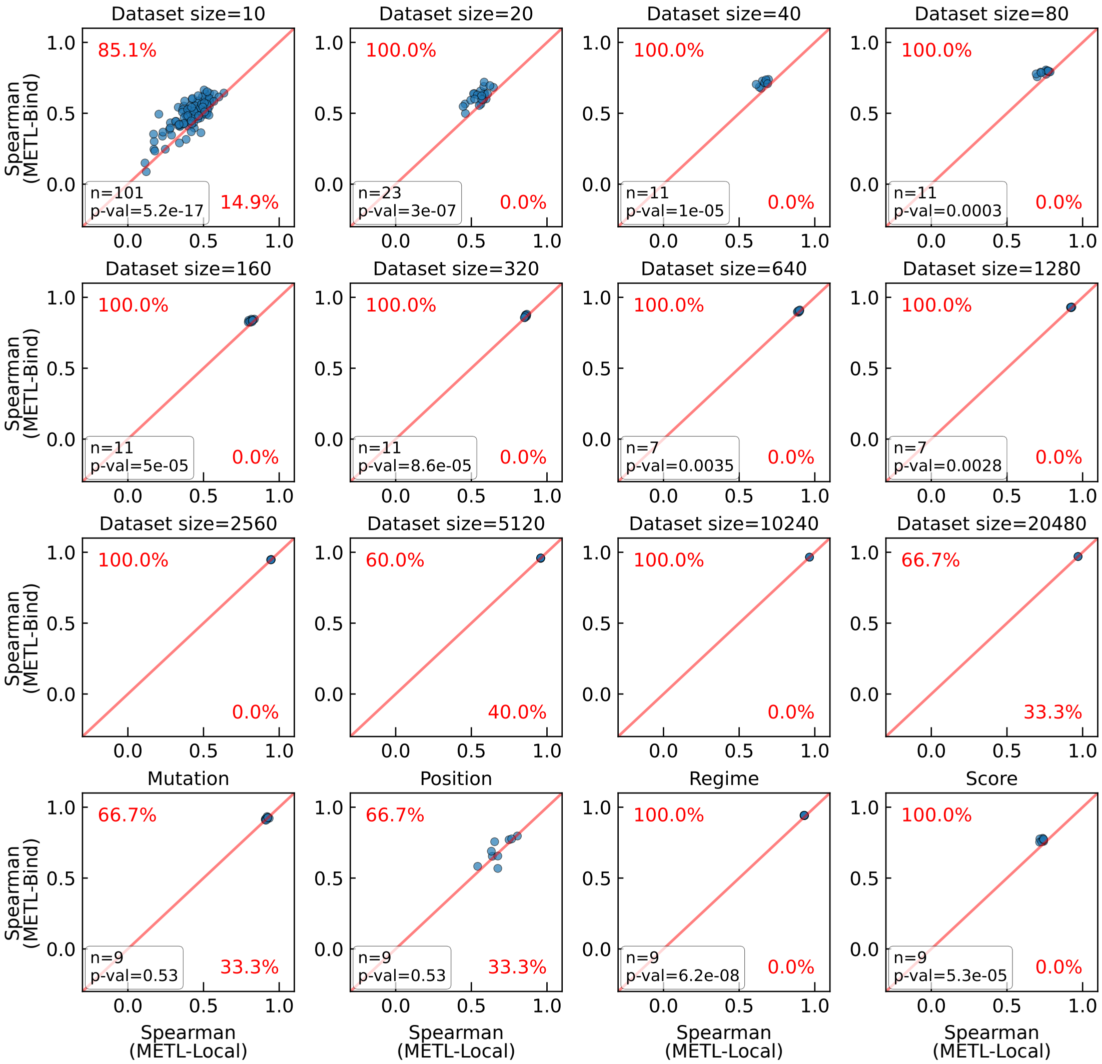

Figure S17. METL-Local and METL-Bind performance for individual training replicates. These scatterplots show the METL-Local and METL-Bind Spearman correlation performance for individual training replicates across different experimental dataset sizes and extrapolation tasks. The text indicates the percentage of replicates where METL-Bind performs better (upper left) or METL-Local performs better (lower right). For dataset sizes with at least 7 replicates ($n \geq 7$), the plots are annotated with the p-value from a paired t-test, which evaluates whether the observed differences in mean performance between the two methods are statistically significant. These statistical tests confirm that METL-Bind's improvements over METL are statistically significant ($p \leq 0.01$) across all training set sizes with $n \geq 7$ and for regime and score extrapolation even though the effect size can be small.

bioRxiv preprint doi: https://doi.org/10.1101/2024.03.15.585128; this version posted January 14, 2025. The copyright holder for this preprint (which was not certified by peer review) is the author/funder, who has granted bioRxiv a license to display the preprint in perpetuity. It is made available under aCC-BY 4.0 International license.

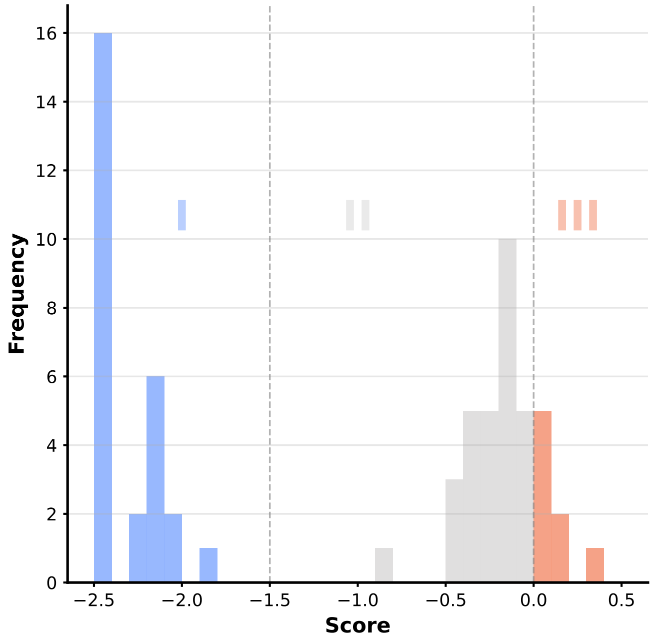

Figure S18. Score distribution of the 64 GFP variants. The 64 GFP variants randomly selected for METL-L-GFP training were split by score into three bins for visualization purposes. The bins were manually defined based on one threshold separating the two main modes of the distribution and another at the wild type score of 0. There are only eight variants in the bin with positive scores. The score represents the variant's brightness.

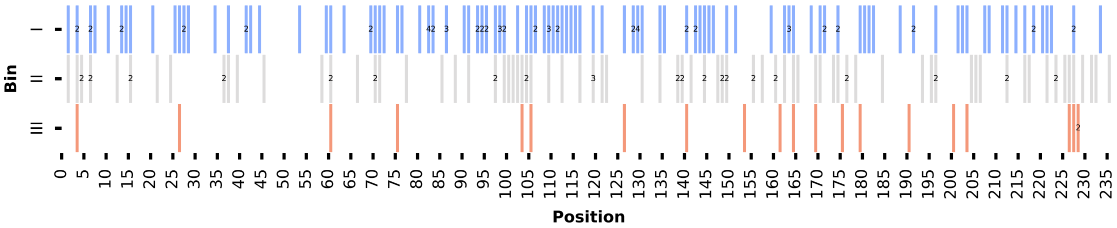

Figure S19. The positions of all 209 unique mutations for the 64 GFP variants The mutations are divided using the same binning procedure and bin labels as Fig. S18. If there were multiple mutations at a position within a bin, it is marked with a numeric label. White indicates that no mutation is present at that position. A bar with no numeric label indicates there is only one mutation present at that position.

bioRxiv preprint doi: https://doi.org/10.1101/2024.03.15.585128; this version posted January 14, 2025. The copyright holder for this preprint (which was not certified by peer review) is the author/funder, who has granted bioRxiv a license to display the preprint in perpetuity. It is made available under aCC-BY 4.0 International license.

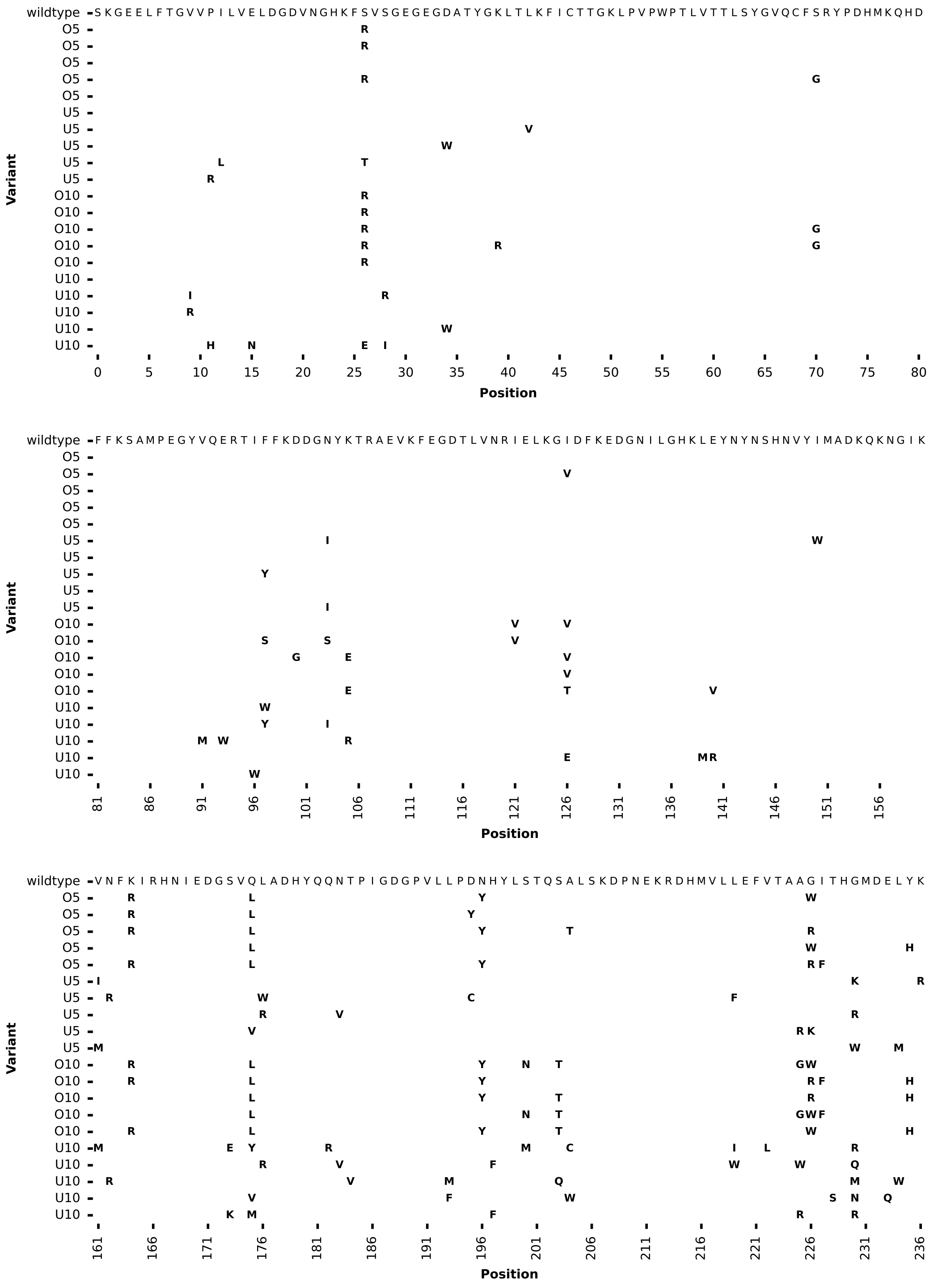

Figure S20. The 20 engineered GFP variants. Each row corresponds to a variant, where if a mutation was made it is shown in bold at the corresponding sequence position. O5 corresponds to Observed 5 mutant designs, U5 Unobserved 5 mutants, O10 Observed 10 mutants, and U10 Unobserved 10 mutants. The wild-type GFP sequence is shown in the top row.

bioRxiv preprint doi: https://doi.org/10.1101/2024.03.15.585128; this version posted January 14, 2025. The copyright holder for this preprint (which was not certified by peer review) is the author/funder, who has granted bioRxiv a license to display the preprint in perpetuity. It is made available under aCC-BY 4.0 International license.

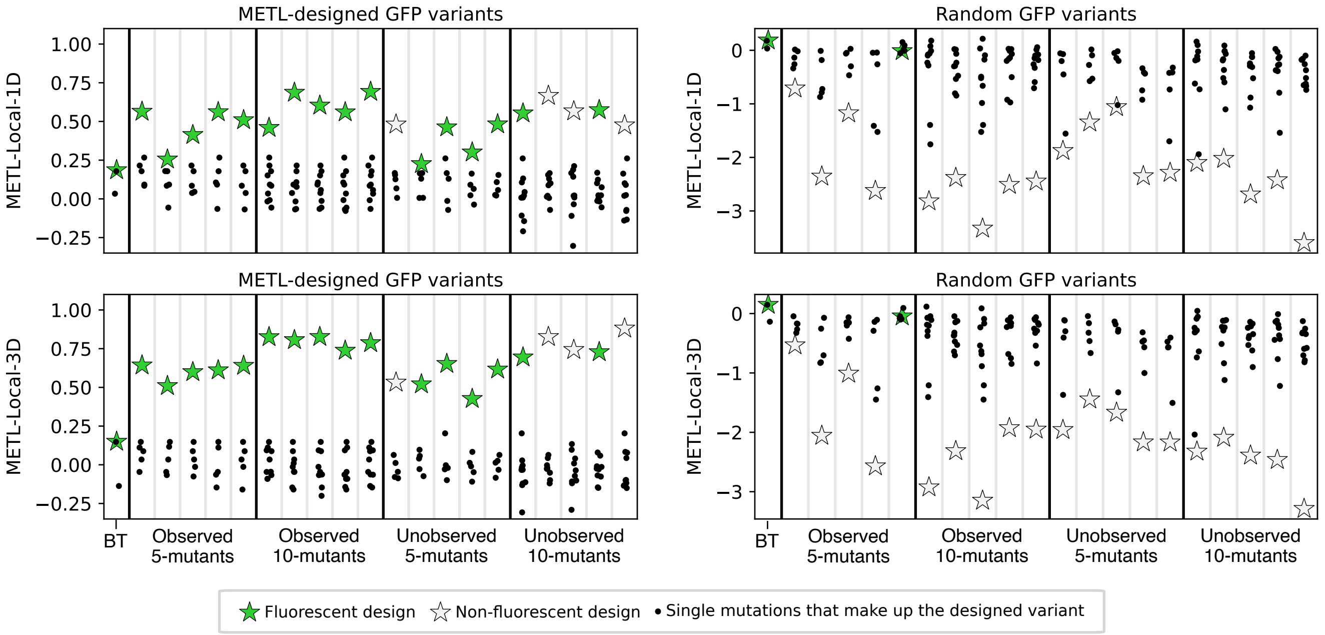

Figure S21. METL predictions for experimentally characterized GFP variants. METL-Local score predictions are shown for both METL-derived and random GFP variants that we experimentally characterized. Predictions from METL-Local models using 1D and 3D relative position embeddings are included. Stars represent predicted scores for the full 5- or 10-mutation variants, whereas black dots indicate the predicted scores for the single-mutation variants that compose these multi-mutation designs. BT represents the METL-Local training set variant with the highest assay score from the DMS dataset.

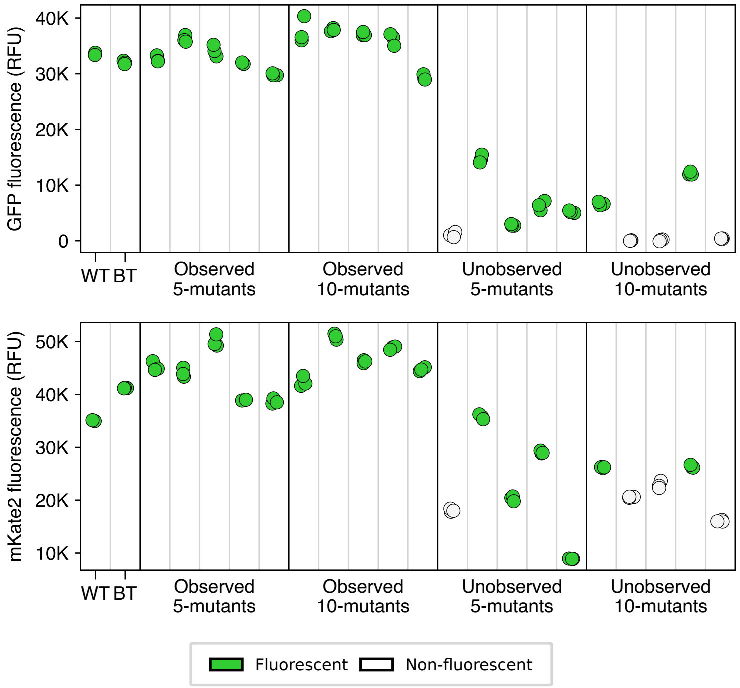

Figure S22. Experimental GFP and mKate2 fluorescence for engineered GFP variants. We expressed the GFP variants as fusion proteins with mKate2. The mKate2 sequence remained constant across the different GFP variants. These plots show GFP and mKate2 fluorescence normalized to optical density and with background fluorescence in negative control subtracted out. The best training set sequence (BT) and the wild-type sequence (WT) are included. Variants are colored according to whether they exhibited GFP fluorescence. Multiple replicates are shown. Because the mKate2 sequence remained constant, variation in mKate2 fluorescence may be due to changes in GFP stability.

bioRxiv preprint doi: https://doi.org/10.1101/2024.03.15.585128; this version posted January 14, 2025. The copyright holder for this preprint (which was not certified by peer review) is the author/funder, who has granted bioRxiv a license to display the preprint in perpetuity. It is made available under aCC-BY 4.0 International license.

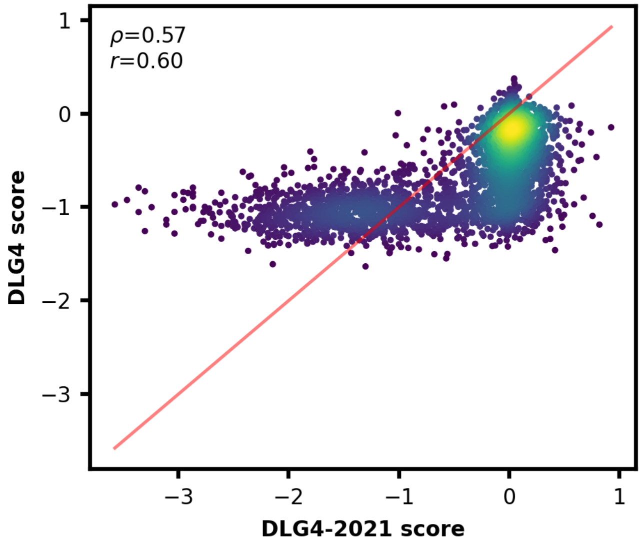

Figure S23. Correlation between DLG4 and DLG4-2021 dataset scores for 3,825 intersecting variants. These datasets both assayed PSD-95 PDZ3 binding to CRIPT, yet they disagree on scores, suggesting differences in methodology. We used DLG4 in our main analysis.

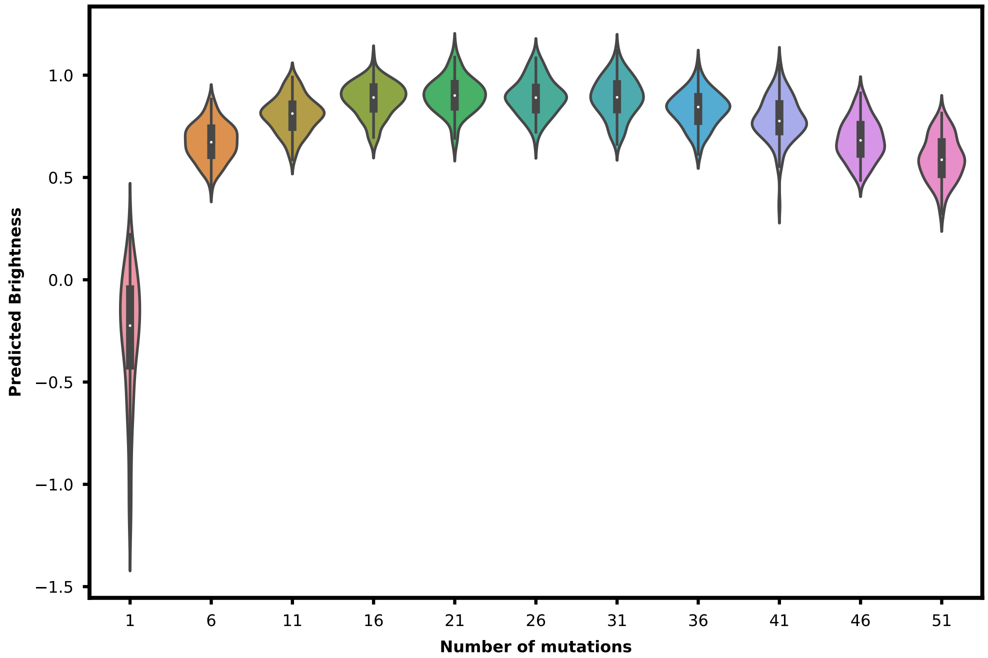

Figure S24. Distribution of METL-L-GFP predicted brightness of best variant found when increasing number of optimized mutations. We ran simulated annealing at different mutational distances from wild type using the same procedure used to design the 20 GFP variants with one exception. Instead of running simulated annealing 10,000 times for each mutation distance, we only ran it 100 times. The distribution of METL-L-GFP predicted brightness scores does not continue to increase as the number of mutations increases.

bioRxiv preprint doi: https://doi.org/10.1101/2024.03.15.585128; this version posted January 14, 2025. The copyright holder for this preprint (which was not certified by peer review) is the author/funder, who has granted bioRxiv a license to display the preprint in perpetuity. It is made available under aCC-BY 4.0 International license.

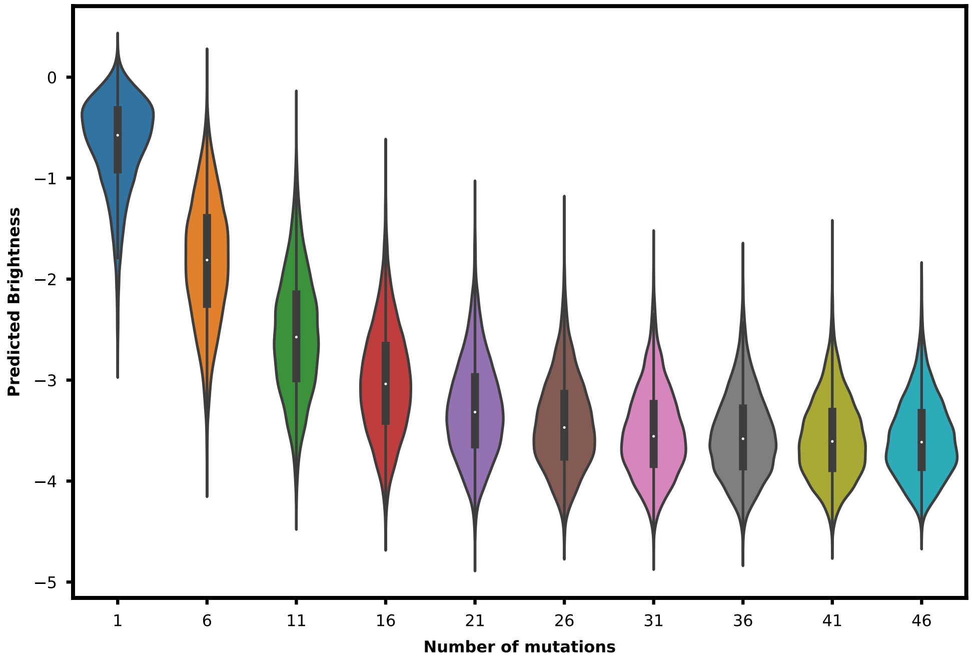

Figure S25. Distribution of METL-L-GFP predicted brightness for increasing number of random mutations. For each mutational distance from wild type, we randomly selected 10,000 variants and calculated their predicted brightness scores. At higher mutational distances, METL-L-GFP predicts lower brightness scores. However, the predicted brightness scores stabilize and do not continue decreasing.

bioRxiv preprint doi: https://doi.org/10.1101/2024.03.15.585128; this version posted January 14, 2025. The copyright holder for this preprint (which was not certified by peer review) is the author/funder, who has granted bioRxiv a license to display the preprint in perpetuity. It is made available under aCC-BY 4.0 International license.

Rosetta score term	Description	Rosetta score term	Description
total_score	REF15	exposed_np_AFIMLWVY	Custom
fa_atr	REF15	exposed_polars	Custom
fa_dun	REF15	exposed_total	Custom
fa_elec	REF15	one_core_each	Custom
fa_intra_rep	REF15	pack	Custom
fa_intra_sol_xover4	REF15	res_count_buried_core	Custom
fa_rep	REF15	res_count_buried_core_boundary	Custom
fa_sol	REF15	res_count_buried_np_core	Custom
hbond_bb_sc	REF15	res_count_buried_np_core_boundary	Custom
hbond_lr_bb	REF15	ss_contributes_core	Custom
hbond_sc	REF15	ss_mis	Custom
hbond_sr_bb	REF15	total_hydrophobic	Custom
lk_ball_wtd	REF15	total_hydrophobic_AFILMVWY	Custom
omega	REF15	total_sasa	Custom
p_aa_pp	REF15	two_core_each	Custom
pro_close	REF15	unsat_hbond	Custom
rama_prepro	REF15	centroid_total_score	Centroid
ref	REF15	cbeta	Centroid
yhh_planarity	REF15	cenpack	Centroid
buried_all	Custom	env	Centroid
buried_np	Custom	hs_pair	Centroid
contact_all	Custom	pair	Centroid
contact_buried_core	Custom	rg	Centroid
contact_buried_core_boundary	Custom	rsigma	Centroid
degree	Custom	sheet	Centroid
degree_core	Custom	ss_pair	Centroid
degree_core_boundary	Custom	vdw	Centroid
exposed_hydrophobics	Custom		

Table S1. Rosetta score terms. The Rosetta score terms used to train METL.

bioRxiv preprint doi: https://doi.org/10.1101/2024.03.15.585128; this version posted January 14, 2025. The copyright holder for this preprint (which was not certified by peer review) is the author/funder, who has granted bioRxiv a license to display the preprint in perpetuity. It is made available under aCC-BY 4.0 International license.

PDB	Len	PDB	Len	PDB	Len	PDB	Len
1A3A	148	1EJ8	140	1IWD	215	1QF9	194
1A6M	151	1EK0	170	1JBK	195	1QJP	171
1A70	97	1F6B	198	1JFU	186	1QL0	241
1AAP	58	1FCY	236	1JFX	217	1R26	125
1ABA	87	1FK5	93	1JKX	212	1ROA	122
1AG6	99	1FL0	171	1JL1	155	1RW1	114
1AOE	192	1FNA	91	1JO0	98	1RW7	243
1ATL	202	1FQT	112	1JO8	58	1RYB	205
1ATZ	189	1FVG	199	1JOS	128	1SMX	96
1AVS	90	1FVK	189	1JVW	167	1SVY	114
1BDO	80	1FX2	235	1JWQ	179	1T8K	77
1BEB	162	1G2R	100	1JYH	157	1TIF	78
1BEH	187	1G9O	91	1K6K	143	1TQG	105
1BKR	109	1GBS	185	1K7C	233	1TQH	247
1BRF	53	1GMI	136	1K7J	206	1TZV	142
1BSG	266	1GMX	108	1KID	203	1VFY	73
1C44	123	1GUU	52	1KQ6	141	1VHU	211
1C52	131	1GZ2	142	1KQR	179	1VJK	98
1C9O	66	1GZC	239	1KTG	138	1VMB	140
1CC8	73	1H0P	182	1KU3	73	1VP6	138
1CHD	203	1H2E	207	1KW4	89	1W0H	204
1CJW	166	1H4X	117	1LM4	194	1WHI	122
1CKE	227	1H98	78	1LO7	141	1WJX	122
1CTF	74	1HDO	206	1LPY	164	1WKC	184
1CXY	90	1HFC	169	1M4J	142	1XDZ	240
1CZN	169	1HH8	213	1M8A	70	1XFF	240
1D0Q	103	1HTW	158	1MK0	97	1XKR	206
1D1Q	161	1HXN	219	1MUG	168	2ARC	164
1D4O	184	1I1J	108	1NB9	147	2CUA	135
1DBX	158	1I1N	226	1NE2	200	2HS1	99
1DIX	208	1I4J	110	1NPS	88	2MHR	118
1DLW	116	1I58	189	1NRV	105	2PHY	125
1DMG	225	1I5G	144	1NY1	240	2TPS	227
1DQG	135	1I71	83	1O1Z	234	2VXN	251
1DSX	87	1IHZ	149	1P90	145	3BOR	237
1EAZ	125	1IIB	106	1PCH	88	3DQG	151
1EJ0	180	1IM5	180	1PKO	139	5PTP	223

Table S2. METL-Global training PDBs. The 148 base PDBs used for the METL-Global simulated pretraining data and their sequence lengths.

	Description	Organism	Molecular function	Selection	Length	Variants	Ref.
GFP	Green fluorescent protein	A. victoria	Fluorescence	Brightness	237	51714	[2]
DLG4-A	Postsynaptic density protein 95 PDZ3 domain	H. sapiens	Synaptic organization	Abundance	84	6976	[3]
DLG4-B	Postsynaptic density protein 95 PDZ3 domain	H. sapiens	Synaptic organization	CRIPT binding	84	8251	[3]
GB1	Protein G B1 domain	Streptococcus sp.	Antibody binding	IgG-Fc binding	56	536084	[4]
GRB2-A	Growth factor receptor-bound protein 2 SH3 domain	H. sapiens	Signaling adaptor	Abundance	56	63366	[3]
GRB2-B	Growth factor receptor-bound protein 2 SH3 domain	H. sapiens	Signaling adaptor	GAB2 binding	56	33441	[3]
Pab1	Pab1 RNA recognition motif (RRM) domain	S. cerevisiae	Poly(A) binding	mRNA binding	75	37710	[5]
PTEN-A	Phosphatase and tensin homolog	H. sapiens	Lipid phosphatase	Abundance	403	4387	[6]
PTEN-E	Phosphatase and tensin homolog	H. sapiens	Lipid phosphatase	Lipid phosphatase activity	403	6564	[7]
TEM-1	TEM-1 β-lactamase	E. coli	Antibiotic hydrolysis	Ampicillin resistance	286	12374	[8]
Ube4b	Ubiquitination factor E4B U-box domain	M. musculus	Ubiquitin activation	Ubiquitin ligase activity	102	88375	[9]

Table S3. Experimental datasets. We evaluated METL on experimental datasets representing proteins of varying sizes, folds, and functions.

bioRxiv preprint doi: https://doi.org/10.1101/2024.03.15.585128; this version posted January 14, 2025. The copyright holder for this preprint (which was not certified by peer review) is the author/funder, who has granted bioRxiv a license to display the preprint in perpetuity. It is made available under aCC-BY 4.0 International license.

Protein	Eval structure	Pretrain struct.	RMSD	TM-score	Identity	Aligned residues	Eval len	Pretrain len
DLG4	6qji_p_trunc_2022.pdb	1g9o_A_p.pdb	1.79	0.87	27%	80	84	91
GRB2	AF-P62993-F1-model_v4_trunc_p.pdb	1jo8_A_p.pdb	1.16	0.90	39%	55	56	58
GRB2	AF-P62993-F1-model_v4_trunc_p.pdb	1i1j_remod_p.pdb	1.34	0.86	23%	56	56	108
TEM-1	AF-Q6SJ61-F1-model_v4_p.pdb	1bsg_p.pdb	1.90	0.85	39%	246	286	266

Table S4. Similarity between METL-Global pretraining structures and the downstream evaluation proteins. We clustered protein sequences and structures and used the RCSB PDB pairwise structure alignment tool to compare the METL-Global pretraining structures with the downstream evaluation proteins. Root-Mean-Square-Deviation (RMSD) is reported in Ångstroms. Template modeling score (TM-score) ranges from 0 to 1, with higher scores indicating stronger similarity. Identity is the percent sequence identity.

Rosetta score term	Description
complex_normalized	InterfaceAnalzyer
dG_cross	InterfaceAnalzyer
dG_cross/dSASAx100	InterfaceAnalzyer
dG_separated	InterfaceAnalzyer
dG_separated/dSASAx100	InterfaceAnalzyer
dSASA_hphobic	InterfaceAnalzyer
dSASA_int	InterfaceAnalzyer
dSASA_polar	InterfaceAnalzyer
delta_unsatHbonds	InterfaceAnalzyer
hbond_E_fraction	InterfaceAnalzyer
hbonds_int	InterfaceAnalzyer
nres_int	InterfaceAnalzyer
per_residue_energy_int	InterfaceAnalzyer
side1_normalized	InterfaceAnalzyer
side1_score	InterfaceAnalzyer
side2_normalized	InterfaceAnalzyer
side2_score	InterfaceAnalzyer

Table S5. Binding score terms. The Rosetta binding score terms, calculated on the GB1-IgG complex structure and used in addition to the standard score terms to train METL-Bind.

bioRxiv preprint doi: https://doi.org/10.1101/2024.03.15.585128; this version posted January 14, 2025. The copyright holder for this preprint (which was not certified by peer review) is the author/funder, who has granted bioRxiv a license to display the preprint in perpetuity. It is made available under aCC-BY 4.0 International license.

ID	Constraint	# Muts	Mutations
1	Observed	5	S26R,K164R,Q175L,N196Y,G226W
2	Observed	5	S26R,I126V,K164R,Q175L,D195Y
3	Observed	5	K164R,Q175L,N196Y,A204T,G226R
4	Observed	5	S26R,S70G,Q175L,G226W,Y235H
5	Observed	5	K164R,Q175L,N196Y,G226R,I227F
6	Unobserved	5	N103I,I150W,V161I,G230K,K236R
7	Unobserved	5	L42V,N162R,L176W,D195C,L219F
8	Unobserved	5	D34W,F97Y,L176R,N183V,G230R
9	Unobserved	5	I12L,S26T,Q175V,A225R,G226K
10	Unobserved	5	P11R,N103I,V161M,G230W,L234M
11	Observed	10	S26R,I121V,I126V,K164R,Q175L,N196Y,S200N,S203T,A225G,G226W
12	Observed	10	S26R,F97S,N103S,I121V,K164R,Q175L,N196Y,G226R,I227F,Y235H
13	Observed	10	S26R,S70G,D100G,K105E,I126V,Q175L,N196Y,S203T,G226R,Y235H
14	Observed	10	S26R,K39R,S70G,I126V,Q175L,S200N,S203T,A225G,G226W,I227F
15	Observed	10	S26R,K105E,I126T,E140V,K164R,Q175L,N196Y,S203T,G226W,Y235H
16	Unobserved	10	F97W,V161M,S173E,Q175Y,Q182R,S200M,A204C,L219I,V222L,G230R
17	Unobserved	10	V9I,S28R,F97Y,N103I,L176R,N183V,H197F,L219W,A225W,G230Q
18	Unobserved	10	V9R,V91M,E93W,K105R,N162R,T184V,L193M,S203Q,G230M,L234W
19	Unobserved	10	D34W,I126E,L139M,E140R,Q175V,L193F,A204W,T228S,G230N,E233Q
20	Unobserved	10	P11H,E15N,S26E,S28I,I96W,S173K,Q175M,H197F,A225R,G230R

Table S6. METL-designed GFP sequences. The METL-designed sequences in the GFP low-N design experiment.

Dataset	Structure acquired from	Notes	Num variants
GFP	RosettaCM		18,681,329
DLG4-2021	PDB: 6QJI	Structure not truncated.	20,270,692
DLG4-A/B	PDB: 6QJI	Structure not truncated. Dataset is based on the DLG4-2021 dataset but contains approximately 2M additional variants to cover additional residues present in the reference sequence.	22,221,845
GB1	PDB: 2QMT		12,556,374
GRB2-A/B	AlphaFold DB: AF-P62993-F1-model_v4	Structure truncated to match DMS sequence.	20,294,793
Pab1	RosettaCM		19,667,539
PTEN-A/E	AlphaFold DB: AF-P60484-F1-model_v4		19,832,384
TEM-1	AlphaFold DB: AF-Q6SJ61-F1-model_v4		19,441,290
Ube4b	RosettaCM		19,734,229

Table S7. Rosetta datasets for METL-Local. Information about the Rosetta datasets used to train the METL-Local source models, including PDB origin and the final number of variants in each dataset. The DLG4-2021 dataset was not used in the main analysis.

	Acquired from	Files / URN / Accession	Variant filtering	Score transformation	Ref.
GFP	Paper supplement	amino_acid_genotypes_to_brightness.tsv	Drop variants with mutations to stop codons	Normalized to WT by subtracting WT score	[2]
DLG4-2021	MaveDB	urn:mavedb:00000053-a	Keep if (inp >= 200) or (inp > 10 and sel >= 1)	N/A	[10]
DLG4-Abundance	NCBI GEO	GSE184042	N/A	Normalized to WT by subtracting WT score	[3]
DLG4-Binding	NCBI GEO	GSE184042	N/A	Normalized to WT by subtracting WT score	[3]
GB1	Paper supplement	mmc2.xlsx	Keep if input_count + sel_count >= 5	Computed from read counts w/ Enrich2 [11]	[4]
GRB2-Abundance	NCBI GEO	GSE184042	N/A	Normalized to WT by subtracting WT score	[3]
GRB2-Binding	NCBI GEO	GSE184042	N/A	Normalized to WT by subtracting WT score	[3]
Pab1	Paper supplement	Supplementary tables 2 and 5	N/A	Converted to log scores by taking log base 2	[5]
PTEN-Abundance	Paper supplement	Supplementary table 3	Keep only missense variants	Normalized to WT by subtracting WT score	[6]
PTEN-Activity	Paper supplement	Supplementary table 2	Keep high-confidence missense; drop NaN scores	N/A	[7]
TEM-1	Paper supplement	mmc2.xlsx	N/A	Converted to log scores by taking log base 2	[8]
Ube4b	MaveDB	urn:mavedb:00000004-a-3	Drop variants with mutations to stop codons	N/A	[9]

Table S8. Experimental dataset preprocessing. This table specifies the experimental datasets used in this study, where we acquired them from, and any filtering or transformations we applied to standardize the dataset format. The DLG4-2021 dataset was not used in the main analysis.

bioRxiv preprint doi: https://doi.org/10.1101/2024.03.15.585128; this version posted January 14, 2025. The copyright holder for this preprint (which was not certified by peer review) is the author/funder, who has granted bioRxiv a license to display the preprint in perpetuity. It is made available under aCC-BY 4.0 International license.

References

1. Rives, A. *et al.* Biological structure and function emerge from scaling unsupervised learning to 250 million protein sequences. *Proc. Natl. Acad. Sci.* **118**, e2016239118. doi:10.1073/pnas.2016239118 (2021).
2. Sarkisyan, K. S. *et al.* Local fitness landscape of the green fluorescent protein. *Nature* **533**, 397–401. doi:10.1038/nature17995 (2016).
3. Faure, A. J. *et al.* Mapping the energetic and allosteric landscapes of protein binding domains. *Nature* **604**, 175–183. doi:10.1038/s41586-022-04586-4 (2022).
4. Olson, C. A., Wu, N. C. & Sun, R. A Comprehensive Biophysical Description of Pairwise Epistasis throughout an Entire Protein Domain. *Curr. Biol.* **24**, 2643–2651. doi:10.1016/j.cub.2014.09.072 (2014).
5. Melamed, D., Young, D. L., Gamble, C. E., Miller, C. R. & Fields, S. Deep mutational scanning of an RRM domain of the Saccharomyces cerevisiae poly(A)-binding protein. *RNA* **19**, 1537–1551. doi:10.1261/rna.040709.113 (2013).
6. Matreyek, K. A., Stephany, J. J., Ahler, E. & Fowler, D. M. Integrating thousands of PTEN variant activity and abundance measurements reveals variant subgroups and new dominant negatives in cancers. *Genome Medicine* **13**, 165. doi:10.1186/s13073-021-00984-x (2021).
7. Mighell, T. L., Evans-Dutson, S. & O'Roak, B. J. A Saturation Mutagenesis Approach to Understanding PTEN Lipid Phosphatase Activity and Genotype-Phenotype Relationships. *The Am. J. Hum. Genet.* **102**, 943–955. doi:10.1016/j.ajhg.2018.03.018 (2018).
8. Gonzalez, C. E. & Ostermeier, M. Pervasive Pairwise Intragenic Epistasis among Sequential Mutations in TEM-1 β-Lactamase. *J. Mol. Biol.* **431**, 1981–1992. doi:10.1016/j.jmb.2019.03.020 (2019).
9. Starita, L. M. *et al.* Activity-enhancing mutations in an E3 ubiquitin ligase identified by high-throughput mutagenesis. *Proc. Natl. Acad. Sci.* **110**, E1263–E1272. doi:10.1073/pnas.1303309110 (2013).
10. Nedrud, D., Coyote-Maestas, W. & Schmidt, D. A large-scale survey of pairwise epistasis reveals a mechanism for evolutionary expansion and specialization of PDZ domains. *Proteins: Struct. Funct. Bioinforma.* **89**, 899–914. doi:10.1002/prot.26067 (2021).
11. Rubin, A. F. *et al.* A statistical framework for analyzing deep mutational scanning data. *Genome Biol.* **18**, 150. doi:10.1186/s13059-017-1272-5 (2017).
